# Consistent features observed in structural probing data of eukaryotic RNAs

**DOI:** 10.1093/nargab/lqaf001

**Published:** 2025-01-30

**Authors:** Kazuteru Yamamura, Kiyoshi Asai, Junichi Iwakiri

**Affiliations:** Department of Computational Biology and Medical Sciences, Graduate School of Frontier Sciences, The University of Tokyo, Kashiwanoha 5-1-5, Kashiwa, Chiba 277-8561, Japan; Department of Computational Biology and Medical Sciences, Graduate School of Frontier Sciences, The University of Tokyo, Kashiwanoha 5-1-5, Kashiwa, Chiba 277-8561, Japan; Department of Computational Biology and Medical Sciences, Graduate School of Frontier Sciences, The University of Tokyo, Kashiwanoha 5-1-5, Kashiwa, Chiba 277-8561, Japan

## Abstract

Understanding RNA structure is crucial for elucidating its regulatory mechanisms. With the recent commercialization of messenger RNA vaccines, the profound impact of RNA structure on stability and translation efficiency has become increasingly evident, underscoring the importance of understanding RNA structure. Chemical probing of RNA has emerged as a powerful technique for investigating RNA structure in living cells. This approach utilizes chemical probes that selectively react with accessible regions of RNA, and by measuring reactivity, the openness and potential of RNA for protein binding or base pairing can be inferred. Extensive experimental data generated using RNA chemical probing have significantly contributed to our understanding of RNA structure in cells. However, it is crucial to acknowledge potential biases in chemical probing data to ensure an accurate interpretation. In this study, we comprehensively analyzed transcriptome-scale RNA chemical probing data in eukaryotes and report common features. Notably, in all experiments, the number of bases modified in probing was small, the bases showing the top 10% reactivity well reflected the known secondary structure, bases with high reactivity were more likely to be exposed to solvent and low reactivity did not reflect solvent exposure, which is important information for the analysis of RNA chemical probing data.

## Introduction

RNA is a major regulatory factor in cells, and elucidating its regulatory mechanisms requires an understanding of the sequence and structure of RNA (1). The roots of this recognition can be found in the 1980s with discoveries such as RNA self-splicing (2) and ribozymes (3). In recent years, messenger RNA (mRNA) vaccines have been commercialized, and it has been shown that RNA structure is deeply involved in its thermal stability and translation efficiency (4). Thus, the importance of understanding RNA structure is increasing.

The structure of RNA can be analyzed using various methods. However, the development of chemical probing of RNA using next-generation sequencing has revolutionized our ability to gather information on RNA structure within living cells (5). RNA chemical probing is performed in three steps: introduce a chemical compound that reacts with specific sites of RNA in cells; sequence the RNA extracted from the cells; and identify the positions of the bases that underwent chemical reactions and calculate the reactivity. Bases with higher reactivity to the chemical compound are inferred to be in a chemical probe-accessible RNA structure that would not be involved in base pair formation or protein binding (5).

There are two main methods for detecting modified bases in RNA chemical probing: the reverse transcription stop profiling method (SEQ), which utilizes the fact that reverse transcription stops at modified bases, and the mutational profiling method (MaP), which utilizes the fact that modified bases are more likely to undergo mutation in reverse transcription (6). The advantages of using chemical probes include that they can be used to detect the *in vivo* structure of RNA and to perform high-throughput structure analysis at the transcriptome scale. Among the methods for RNA structure determination using chemical probes, the standard methods are the dimethyl sulfate (DMS) method and selective 2′-hydroxyl acylation analyzed by the primer extension (SHAPE) method (7). Both methods have been used in a number of studies to investigate RNA structure *in vivo* at the transcriptome scale using eukaryotic cells, including human (8–10), mouse (10), *Arabidopsis* (11), rice (12) and yeast (8,9). The results of these RNA chemical probing experiments can be used to analyze the secondary and tertiary structures of RNA as well as the molecular interactions that occur *in vivo* (6). Secondary structure is represented by dot-and-bracket notation, with a dot indicating an unpaired and a bracket indicating a base-paired structure, and chemical probing data can be used as input for the secondary structure prediction algorithms (13). For example, Deigan *et al.* proposed adjustments to the pseudo-energies for bases involved in base pairing (14), Zarringhalam *et al.* proposed pseudo-energy adjustments including loop structures (15) and Washietl *et al.* proposed a method of reflecting experimental data by maximizing the consistency of energy parameters of substructures with experimental data (16). More recently, Wayment-Steele *et al.* improved the accuracy of secondary structure prediction using chemical probing data indirectly by multi-task learning of the model (17). In tertiary structure prediction, chemical probing data have also been used to predict the solvent-accessible surface area (18). RNA chemical probing has been recognized as a valuable tool for understanding cellular RNA structure.

However, it is worth noting that there have been reported biases in the results of chemical probing experiments, including reports that C2′-endo and certain small loops are associated with higher reactivity, and reports that the relative orientation of the 3′-phosphodiester relative to the 2′-OH group is associated with reactivity (19–25). Therefore, it is crucial to be aware of the biases present in SHAPE and DMS experimental data, as well as reliable reactivity thresholds, for the accurate interpretation of secondary and tertiary structures *in vivo*. Despite the accumulation of a large amount of RNA chemical probing data and the increasing importance of utilizing RNA structure data for advancements, such as in mRNA vaccines, the barrier for most experimental biologists to grasp and utilize large-scale data remains very high (26).

Exploratory data analysis (EDA) can be used to visualize the overall picture of data and summarize the key features (27). By summarizing the dataset using statistical measures, tables or graphs, EDA plays a crucial role in understanding data quality, formulating hypotheses and considering analysis methods. EDA has been applied to various biological data, such as genome-wide association studies and gene expression data, and the understanding of data characteristics has been utilized for research planning (28,29). Although individual data analysis is performed for each RNA chemical probing experiment, comprehensive EDA of data from multiple studies has not been conducted. Possible EDA approaches for multiple chemical probing experiments include exploring the proportion of reactive bases across various RNA chemical probing experiments and the identification of reactivities that strongly correlate with base pairing. Visualizations derived from these analyses can provide valuable insights for researchers who use transcriptome-scale RNA chemical probing data, as well as those seeking to apply machine learning to RNA chemical probing data.

In this study, we performed EDA of RNA chemical probing data generated at the transcriptome scale for eukaryotic cells. Our results demonstrate that, regardless of the experimental group, only a small number of bases were modified by the probes, the top 10% of reactivities well reflected the secondary structure and nucleotides with high reactivities were more likely to be exposed to solvent on the three-dimensional (3D) structure, while low reactivities did not reflect the solvent exposure status. These were common features in diverse eukaryotic cells from different experimental groups and provide important information for the use of existing RNA chemical probing data to obtain hints about the 3D structure, as well as the secondary structure, of an RNA of interest.

## Materials and methods

### Data download and mapping

In this study, publicly available chemical probing data for eukaryotes [human, mouse, *Saccharomyces cerevisiae* (yeast), rice and *Arabidopsis thaliana*]were obtained from the National Center for Biotechnology Information Sequence Read Archive (SRA). These data are summarized in Tables 1–3. The ribosomal RNAs (rRNAs) and mRNA IRES (internal ribosome entry site) sequences were downloaded from the RNAcentral (30) and IRESbase (31), respectively.

**Table 1.** Summary of RNA chemical probing data and mapping results to 18S rRNA

Species	Method	Detection	Use background	Mapped fragments	Average depth	SRA run ID	18S rRNA (RNAcentral)	AUC
Human	DMS	RT stop	No	20 773 031	412 419	SRR1058055	URS0000726FAB_9606	0.67
Rice	DMS	RT stop	Yes	86 344	4177	SRR5297221, SRR5297223	URS000083F611_39 947	0.61
Yeast	DMS	RT stop	Yes	10 810 231	294 116	SRR815612, SRR815617	URS0002311A0A_559 292	0.67
*Arabidopsis*	DMS	RT stop	Yes	8 279 303	181 010	SRR933551, SRR933552	URS000008172F_3702	0.68
Human	DMS	Mutation	No	3 165 179	85 369	SRR3929619, SRR3929620	URS0000726FAB_9606	0.69
Yeast	DMS	Mutation	No	1 892 759	52 233	SRR3929621, SRR3929626	URS0002311A0A_559 292	0.67
Human	SHAPE (NAI-N3)	RT stop	Yes	13 843 457	256 246	SRR7618815, SRR7618817	URS0000726FAB_9606	0.66
Mouse	SHAPE (NAI-N3)	RT stop	Yes	821 162	20 293	SRR7618833, SRR7618835	URS00005B0A54_10 090	0.65
Human	SHAPE (2A3)	Mutation	Yes	490 310	20 039	SRR12235552,SRR12235545	URS0000726FAB_9606	0.65

RT, reverse transcription. AUC was calculated using probe (+) data.

The downloaded data were converted to FASTQ format using the following command with fastq-dump from SRA-Tools (version 2.11.0): fastq-dump --stdout [input file] > [output file].

Quality control was performed using the following command with fastp (version 0.23.3): fastp -i [input file] -o [output file] -w 2 -q 25 -u 20.

Data with duplicated barcode sequences were removed using the method described by the Sun *et al.* (10).

The reference sequence for mapping was built using Bowtie2-build (version 2.2.4) with the following sequence of rRNA: bowtie2 -U [input fastq file] -S [output file] -x [transcriptome build file] --non-deterministic –time.

### Calculation and analysis of reactivity

After the read mapping to reference RNAs, the mapped positions with a coverage of over 500 were included in the analysis. For mRNA analysis, only mRNAs with at least 50 bases with a coverage of over 500 were considered for analysis.

In the MaP, the mutation rate of a base at a given position in the RNA sequence was calculated using the following equation:


\begin{equation*}{\rm Mrate}{\mathrm{\ }} = {\mathrm{\ }}{\rm Mnum}{\mathrm{\ }}/{\mathrm{\ }}{\rm Mdepth},\end{equation*}

where Mrate is the mutation rate of a base at a given position, Mnum is the total number of bases that were mutated at a given position and Mdepth is the total number of bases that were read at a given position.

In the SEQ, the stop rate of a base at a given position in the RNA sequence was calculated using the following equation:


\begin{equation*}{\rm STrate}{\mathrm{\ }} = {\mathrm{\ }}{\rm STnum}{\mathrm{\ }}/{\mathrm{\ }}{\rm STdepth},\end{equation*}

where STrate is the stop rate of a base at a given position, STnum is the total number of bases at which reverse transcription was stopped and STdepth is the total number of bases at which reverse transcription was stopped.

For the several SHAPE/DMS experiments, when the background experimental data without using the chemical probe were available, background-adjusted reactivity was calculated as follows:


\begin{equation*}{\rm STrate}\left( {{\mathrm{chem}}\_{\mathrm{probe}} + } \right){\mathrm{\ }}-{\mathrm{\ }}{\rm STrate}\left( {{\mathrm{chem}}\_{\mathrm{probe}} - } \right).\end{equation*}

In the case of SHAPE experiments, all bases (AUGC) were considered for the calculation. In the case of DMS experiments, only adenine and cytosine were considered for the calculation.

The receiver operating characteristic (ROC) curves were drawn for bases with high reactivity that were considered positive, and true values were assigned to cases in which the dot–bracket data were dots. ROC and area under the curve (AUC) were calculated using scikit-learn (version 1.3.2; https://scikit-learn.org/) (32). The graphs were drawn using Matplotlib (version 3.8.1; https://matplotlib.org/) (33).

### Calculation of base pair probabilities

RNAfold (Vienna package 2.4.6) (34) was used to calculate the base pair probabilities. The probability that the *i*th base pairs with any other base was calculated as the sum of the base pair probabilities between the *i*th base and all other bases. We used these marginalized probabilities for subsequent analyses.

### Calculation of solvent accessibility

Solvent accessibility was calculated using PyMOL (version 2.5.0) with a probe radius of 3 Å. The mouse (ID: 7CPU), human (ID: 8JDK) and yeast (ID: 8CCS) ribosome structures were obtained from the Protein Data Bank (PDB; https://www.rcsb.org/). For the missing residues in 3D structure, their reactivity data were excluded from the evaluation because solvent accessibility could not be calculated for them. High-resolution ribosome structures for *Arabidopsis* and rice had not been available in PDB; therefore, the analysis was not conducted for these species.

## Results and discussion

### Mapping of transcriptome-scale eukaryotic DMS/SHAPE data to 18S/25S/28S rRNA

In the DMS and SHAPE data, it is considered that bases with high reactivity are in an unpaired structure, and it is important to know the criteria for determining which reactivity values indicate an unpaired structure. By comparing known RNA structure data with available RNA chemical probing data, the relationship between high reactivity and structure can be investigated. Reactivity calculation methods are often optimized for the methodology of each research group; therefore, they may differ between groups in RNA chemical probing experiments. These calculation methods are used in the RASP (RNA Atlas of Structure Probing) database (35), which houses a large collection of RNA chemical probing data. It is useful to know the reactivity optimized for each method. However, it is difficult to perform comparisons between different sets of experimental data, and it is also impossible to obtain information on read depth. Therefore, we downloaded transcriptome-scale eukaryotic RNA chemical probing data generated by next-generation sequencing. rRNA accounts for ∼80% of the transcriptome (36); poly(A) selection or rRNA depletion is often employed. Despite these treatments, rRNA can still represent more than a few percent of sequencing reads (36). In several chemical probing studies, rRNA was used for performance evaluation (9,11,12,36). In this study, we also mapped the sequencing reads to 18S/25S/28S rRNA to facilitate comparisons across various RNA chemical probing data. Across all experimental data, a sufficient number of reads (>4177) were mapped to rRNA (Tables 1 and 2).

**Table 2.** Summary of RNA chemical probing data and mapping results to 25/28S/ rRNA

Species	Method	Detection	Use background	Mapped fragments	Average read depth	SRA run ID	25S/28S rRNA (RNAcentral)	AUC
Human	DMS	RT stop	No	14 881 045	109 173	SRR1058055	URS0000E60B81_9606	0.60
Yeast	DMS	RT stop	Yes	19 652 015	284 678	SRR815612, SRR815617	URS000061F377_559 292	0.67
Human	DMS	Mutation	No	6 207 899	61 240	SRR3929619, SRR3929620	URS0000E60B81_9606	0.63
Yeast	DMS	Mutation	No	3 900 862	57 464	SRR3929621, SRR3929626	URS000061F377_559 292	0.57
Human	SHAPE (NAI-N3)	RT stop	Yes	46 915 615	213 292	SRR7618815, SRR7618817	URS0000E60B81_9606	0.69
Human	SHAPE (2A3)	Mutation	Yes	426 523	6419	SRR12235552, SRR12235545	URS0000E60B81_9606	0.76

RT, reverse transcription. AUC was calculated using probe (+) data.

### Distribution of mutation/stop rate in RNA chemical probing data

**Figure 1.**
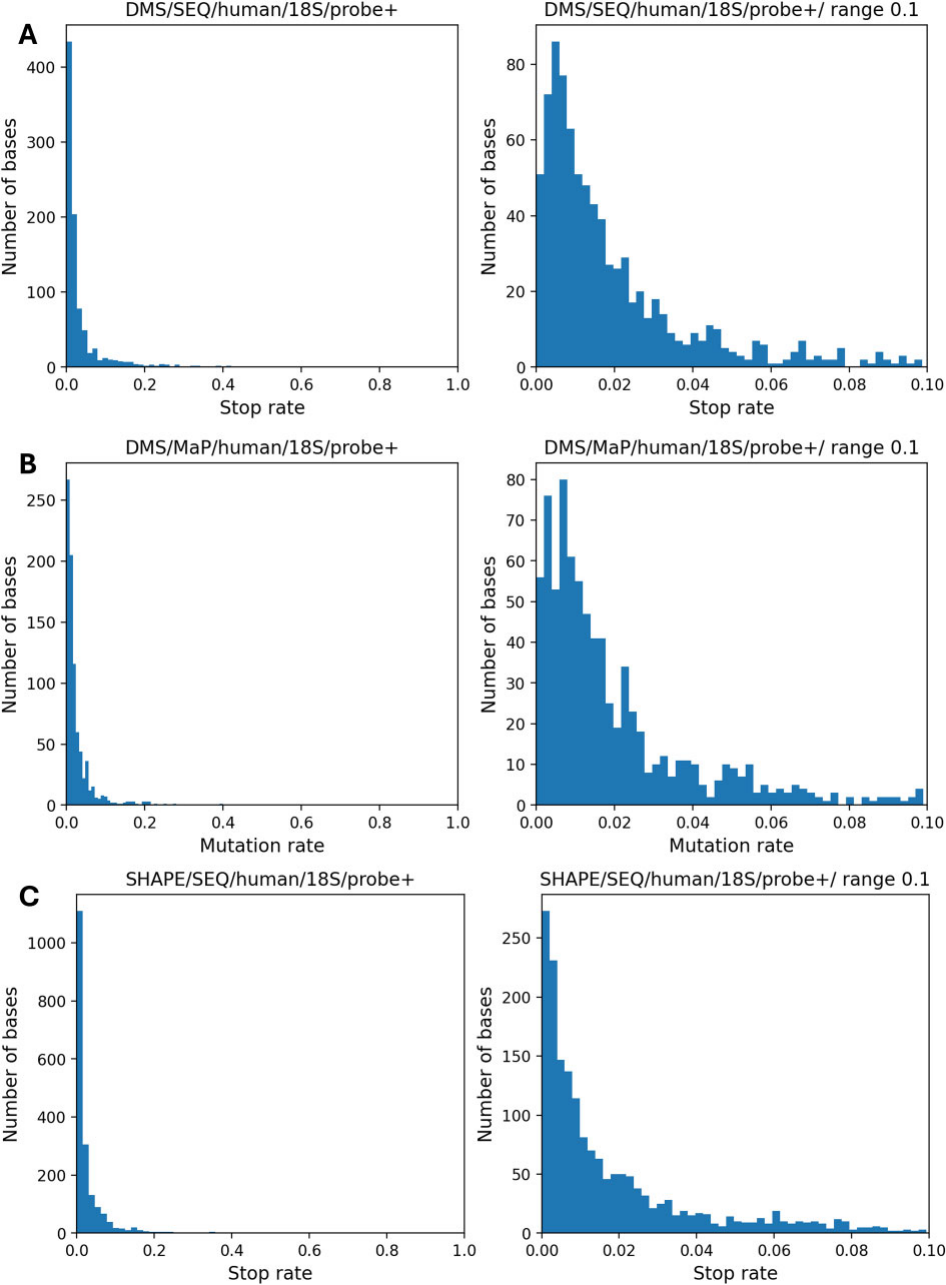
Histograms of the stop/mutation rate in RNA chemical probing experiments. The read data of RNA chemical probing experiments were mapped to 18S/25S/28S rRNA, and the reverse transcription stop and mutation rates were calculated. The horizontal axis represents the stop or mutation rate, while the vertical axis indicates the number of bases associated with the stop or mutation rate. The left-side panel displays a histogram in the range of 0–1.0, while the right-side panel is limited to the range of 0–0.1 for the same histogram. The mutation rate was calculated by dividing the number of specific bases with mutations by the total number of times that base was read. Similarly, the stop rate was calculated by dividing the number of specific bases at which reverse transcription was halted by the total number of times that base was read. (**A**) Data from human DMS stop profiles (8). (**B**) Data from human DMS mutation profiles (9). (**C**) Data from human SHAPE stop profiles (10). Histograms from other experiments are presented in Supplementary Figure S1.

Chemical probing experiments can be divided into two types: those that use chemically modified nucleotides to terminate reverse transcription or to introduce mutations during reverse transcription. To determine the chemical modification rates, the stop/mutation rates were calculated for each type of experiment and their distribution was analyzed (Figure 1, Supplementary Figure S1). It is expected that regions not forming base pairs will have high stop/mutation rates, while regions forming base pairs will have low rates. The percentage of unpaired bases in the secondary structure of 18S/25S/28S rRNA registered in RNAcentral (https://rnacentral.org/) for the studied species was between 38% and 50%. In an ideal experiment, a histogram would show two peaks: one for the well-reacting unpaired bases (∼50%) and one for the nonreacting paired bases (∼50%). However, in all experiments, nearly 90% of bases showed a stop or mutation rate near zero. The percentage of bases with a stop/mutation rate of ≥10% was 8.1%, even in the study with the highest data. It has been reported that SHAPE experiments do not tend to show two peaks in reactivity (37), and this tendency has been found to be the same in all transcriptome-scale experiments, including DMS experiments. The biased reactivity of SHAPE has been reported (19,21,23,25,38), but the present results suggest that biased reactivity is also observed in chemical probing experiments using DMS. Specific RNA structures are reported to be associated with high SHAPE reactivity (38,39); perhaps, DMS is also affected by specific structures.

RNA chemical probing experiments are typically performed based on a criterion called single-hit kinetics, where only one modification occurs per ∼200 nucleotides (40). The bias in reactive bases could be affected by single-hit kinetics. Studies have shown that longer probe reaction times lead to the detection of more loops (41); therefore, longer reaction times may enable the detection of more accessible structures for the chemical probe. However, excessively long reaction times may lead to over-modification by probes, which raises concerns about artificial structural changes and introduces a 5′ positional bias in detection efficiency in reverse transcription profiles due to modifications at closely spaced nucleotides.

When comparing the mutation/stop rate between unpaired and paired bases, the rate is higher for unpaired bases, but there is some overlap in the distribution (Supplementary Figure S6). This suggests that chemical probing data could help us to distinguish base-pairing states in RNA structures. However, the substantial overlap indicates that it may be challenging to achieve a high degree of separation between these structures.

### Analysis of the reactivity distribution of highly solvent-exposed bases

Ribosomes are large molecules with both solvent-exposed and buried regions. Several chemical probing studies have limited their performance evaluation to highly solvent-exposed bases in rRNA, using known structural information (8,9,42). Following this approach, we generated histograms focusing on highly solvent-exposed bases (Supplementary Figure S1M–S). We used ribosomes with high-resolution 3D structures, including mouse (PDB ID: 7CPU) (43), yeast (PDB ID: 8CCS) (44) and human (PDB ID: 8JDK) (45). The structures were downloaded from PDB, and the solvent accessibility of each ribosomal small subunit was calculated (see the ‘Materials and methods’ section). The reactivity distribution of highly solvent exposed regions is almost similar to that of the entire structures shown in Figure 1. This implies that it may be challenging to separate paired and unpaired structures solely based on reactivity data. It should be noted that the ratio of unpaired to paired bases within the solvent-accessible regions is not different from that of the entire ribosome structure.

### In reverse transcription stop experiments, excluding control data can increase pairedness position prediction accuracy

Higher-order RNA structures can be layered into secondary and tertiary structures. To evaluate the accuracy of base pairing prediction in secondary structure using mapped chemical probing data, dot–bracket data of 18S/25S/28S rRNA for each species were downloaded from RNAcentral (Tables 1 and 2).

**Figure 2.**
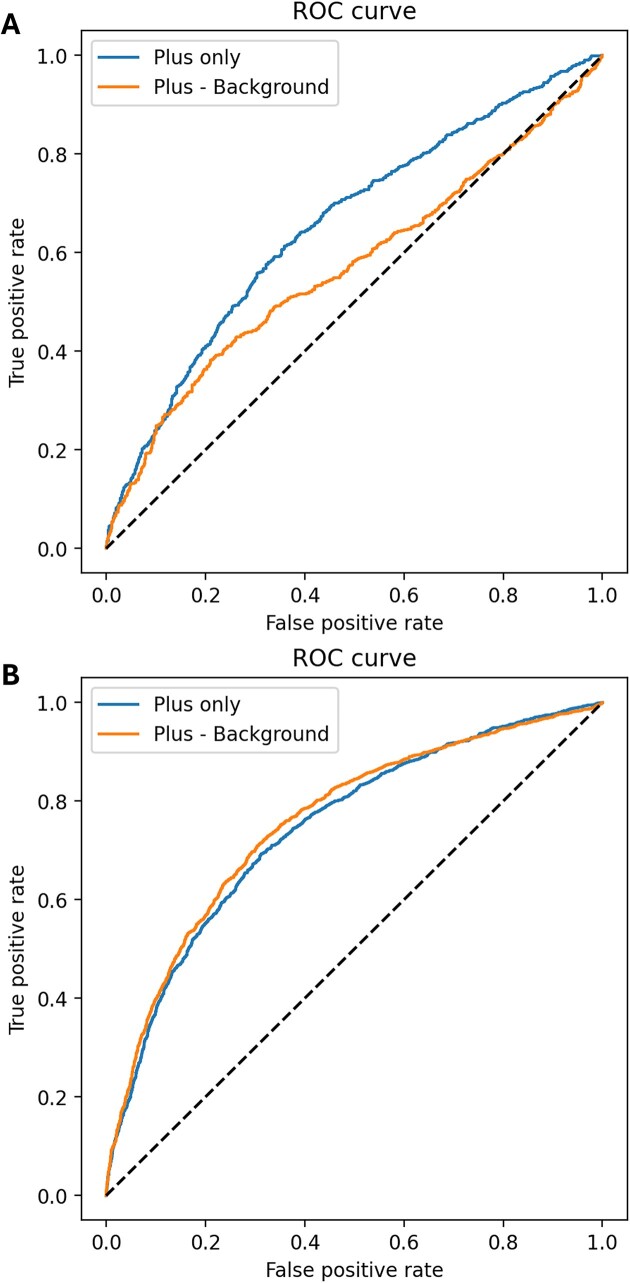
Comparison of ROC curves with and without background subtraction in chemical probing experiments. (**A**) Human data from SHAPE/SEQ (10) were mapped to 18S rRNA. (**B**) Human data from SHAPE (2A3)/MaP (42) were mapped to 28S rRNA, and the ROC curves were plotted. When reactivity is high, it is considered positive, and when the dot–bracket data correspond to a dot, it is labeled as true. The "Plus - Background" represents the ROC curve calculated using background data for reactivity, while the "Plus only" represents the ROC curve calculated without background data for reactivity. For details on the reactivity calculation method, refer to the ‘Materials and methods’ section. Other data can be found in Supplementary Figure S2.

**Figure 3.**
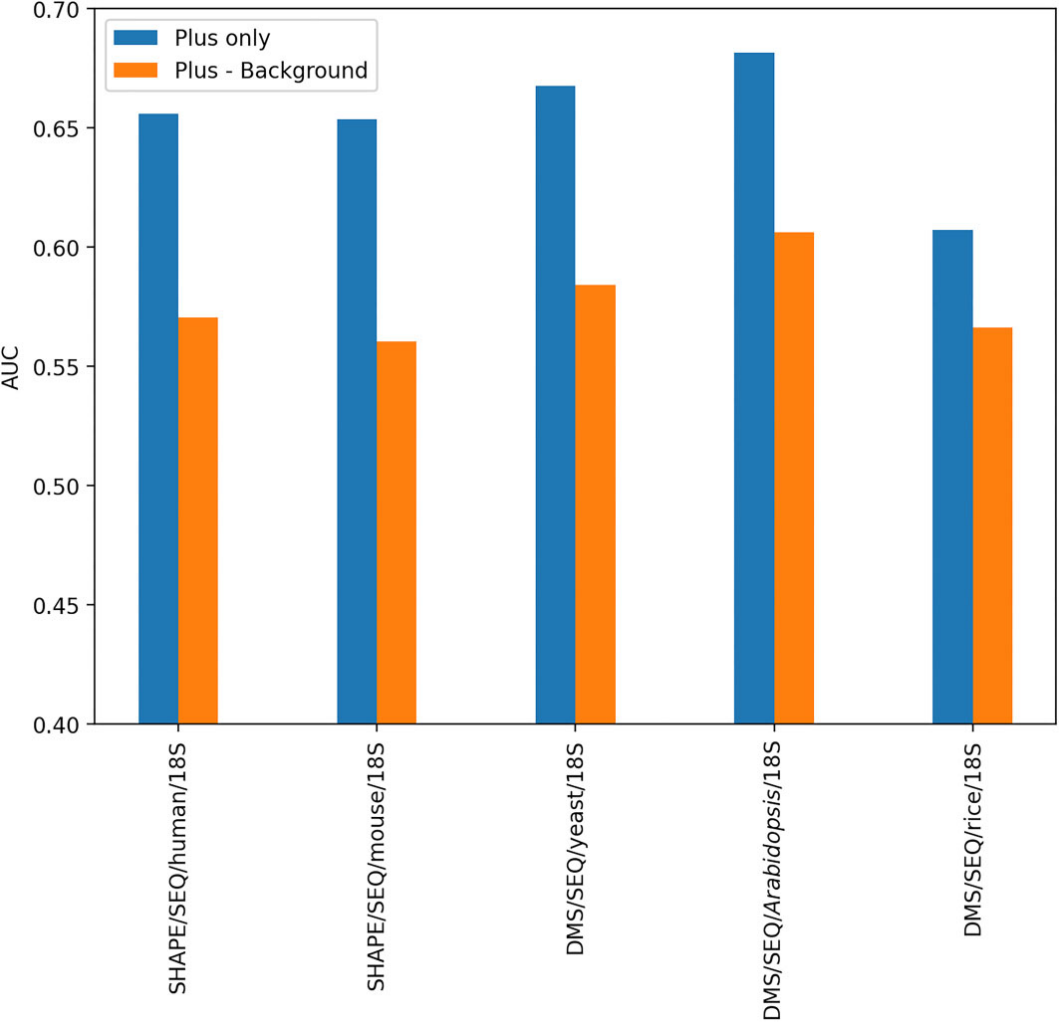
A comparison of whether to use background data to calculate the AUC of the reverse transcription stop profile. When mapping RNA chemical probing data to 18S rRNA and calculating reactivity, a comparison was made between using and not using background data. The "Plus only" represent the AUC value for reactivity calculated without background data, while the "Plus - Background" represent the AUC value when background data were incorporated. AUC was determined by using the secondary structure of 18S rRNA, considering bases with high reactivity as positive and base pairs as true, and then computing the ROC curve.

In RNA chemical probing experiments, there are two main approaches to calculate reactivity: one is calculating the difference between the RNA sequencing data extracted from cells that have (plus) and have not (background) been treated with RNA modification reagents, and the other approach is using only the signal data (plus) obtained with modification reagents (Tables 1 and 2). In several experiments, when both positive and background signals were available, the accuracy of reactivity calculated only from the positive data can be verified. To compare the accuracy of reactivity derived from only positive data and that of reactivity calculated by subtracting background data from positive data, the accuracy of pairedness prediction was compared using ROC curves (Figure 2 and Supplementary Figure S2) and AUC. As a result, the AUC was improved for all data examined when background data were not used to calculate reactivity (Figure 3). The AUC of the *in vivo* SHAPE (NAI) data for *Escherichia coli* ribosomes from Marinus *et al.* (42) was 0.625. This is similar to the AUCs we obtained for SHAPE/SEQ/mouse (0.654) and SHAPE/SEQ/human (0.656), indicating that we achieved a comparable level of analysis. The mutation profiling experiments of Homan *et al.* yielded sufficiently high mutation rates compared to the background; hence, they did not utilize background data (46). Because the mutation profiling experiments of Zubradt *et al.* also exhibited a low correlation of background signals between replicates, they calculated reactivity without using background data (9). The results shown in Supplementary Figure S1 also suggest that background data may not improve the accuracy of reverse transcription stop profiles, as there was no significant difference in the mutation rate and stop rate histograms. Similar to mutation profiling experiments without background data, the results suggest that background signals in reverse transcription stop experiments would be variable and random, introducing noise. Therefore, we have used data without background for reactivity in the following analyses.

Recently, a new SHAPE mutational profiling experiment has been proposed, which involves using a new SHAPE reagent (2A3) and optimizing reverse transcriptase (42). The SHAPE-MaP using 2A3 reagent successfully detected rRNA base pairs more accurately compared to the experiments using other reagents. We also obtained the probing data from HEK293 cells using the 2A3 reagent and conducted a performance evaluation of reactivity using human ribosome. In our evaluation using 28S rRNA, SHAPE-MaP (2A3) also achieved the highest AUC (0.76) (Figure 2B and Table 2). These results suggest that the new SHAPE-MaP experimental protocol using the 2A3 reagent is more accurate in detecting RNA structures. It should be noted that the AUC for the 28S data was 0.76, slightly lower than what was reported in the 2A3 paper. This difference is due to whether the evaluation is limited to solvent-accessible residues or uses the entire structure. Although our evaluation did not limit the assessment to solvent-accessible residues, it is consistent in achieving a higher AUC compared to other experiments.

### Evaluation of RNA chemical probing data using mRNA IRES structure

Evaluation of chemical probing experiments often relies on rRNA (9,11,12,36). However, many researchers have a stronger interest in determining the secondary structures of mRNAs or noncoding RNA. To compare the accuracy of chemical probing experiments for rRNA, as shown in the previous section, with that for mRNA, we used the IRES as a reference. IRESs are well-studied mRNA regions that initiate translation without the 5′ cap structure (47). Their structures and sequences are cataloged in IRESbase (31). To investigate differences between mRNA and rRNA probing data, we mapped the human RNA chemical probing data to IRES sequences obtained from IRESbase (Table 3). We focused on mRNAs with >50 bases and a base density >500. Although SHAPE-MaP (2A3) data also include human mRNA, the data were not included in this analysis as the maximum number of mapped fragments was only 108, which was not sufficient.

**Table 3.** Summary of human RNA chemical probing data mapped to IRES sequence

Species	Method	Detection	Mapped fragments	Average read depth	IRES ID	Gene symbol	AUC
Human	SHAPE (NAI-N3)	RT stop	9590	395	hsa_ires_00184.1	HIST1H1C	0.65
Human	SHAPE (NAI-N3)	RT stop	14 452	419	hsa_ires_00268.1	HIST1H3H	0.84
Human	SHAPE (NAI-N3)	RT stop	1836	358	hsa_ires_00270.1	RPL28	0.67
Human	SHAPE (NAI-N3)	RT stop	2199	431	hsa_ires_00551.1	RPL29	0.72
Human	DMS	RT stop	7007	1462	hsa_ires_00022.1	BSG	0.74
Human	DMS	RT stop	17 127	3637	hsa_ires_00151.1	S100A4	0.55
Human	DMS	RT stop	7559	1596	hsa_ires_00238.1	RPS13	0.41
Human	DMS	RT stop	20 041	4189	hsa_ires_00270.1	RPL28	0.56
Human	DMS	RT stop	4915	1046	hsa_ires_00294.1	MT2A	0.64
Human	DMS	RT stop	4050	842	hsa_ires_00419.1	TUBB6	0.67
Human	DMS	RT stop	16 381	3549	hsa_ires_00482.1	TAGLN	0.65
Human	DMS	RT stop	3910	819	hsa_ires_00521.1	FN1	0.53
Human	DMS	RT stop	11 301	2290	hsa_ires_00570.1	RPL21	0.63
Human	DMS	RT stop	23 617	4136	hsa_ires_00674.1	FTH1	0.63
Human	DMS	Mutation	5248	1507	hsa_ires_00184.1	HIST1H1C	0.6
Human	DMS	Mutation	6668	1915	hsa_ires_00238.1	RPS13	0.71
Human	DMS	Mutation	1971	566	hsa_ires_00268.1	HIST1H3H	0.68
Human	DMS	Mutation	2786	801	hsa_ires_00570.1	RPL21	0.57

RT, reverse transcription. AUC was calculated using probe (+) data.

**Figure 4.**
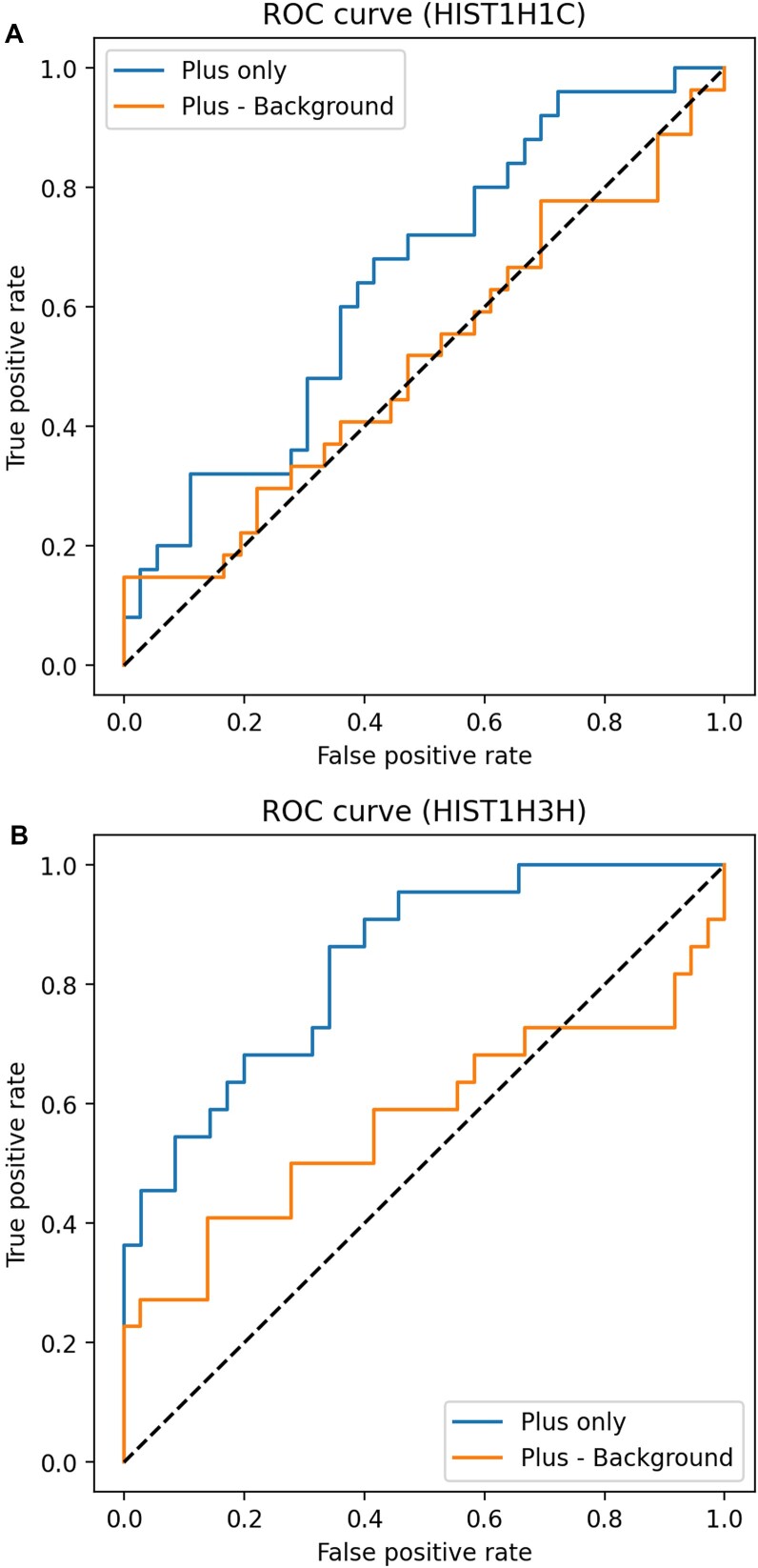
ROC curves generated from SHAPE/SEQ/human data (10) for IRESs. (**A**) Results for IRES ID hsa_ires_00184.1, Gene Symbol HIST1H1C from the IRESbase. (**B**) Results for IRES ID hsa_ires_00268.1, Gene Symbol HIST1H3H from the IRESbase. When reactivity is high, it is considered positive, and when the dot–bracket data correspond to a dot, it is labeled as true. The "Plus - Background" represents the ROC curve calculated using background data for reactivity, while the "Plus only" represents the ROC curve calculated without background data for reactivity. For details on the reactivity calculation method, refer to the ‘Materials and methods’ section. Other AUC data can be found in Table 3.

To assess the agreement between IRESbase structures and chemical probing data, we generated ROC curves (Figure 4 and Table 3). Similar to our findings with rRNA, the average AUC for mRNA IRESs was 0.64, which is similar to the average AUC of 0.66 for rRNA. These results suggest that the performance of chemical probing does not vary across different RNA types. Additionally, our results suggest that accurate structural information of mRNAs could be obtained from chemical probing data, if sufficient read coverage is achieved for mRNAs.

### Data with high reactivity better reflect secondary structures

**Figure 5.**
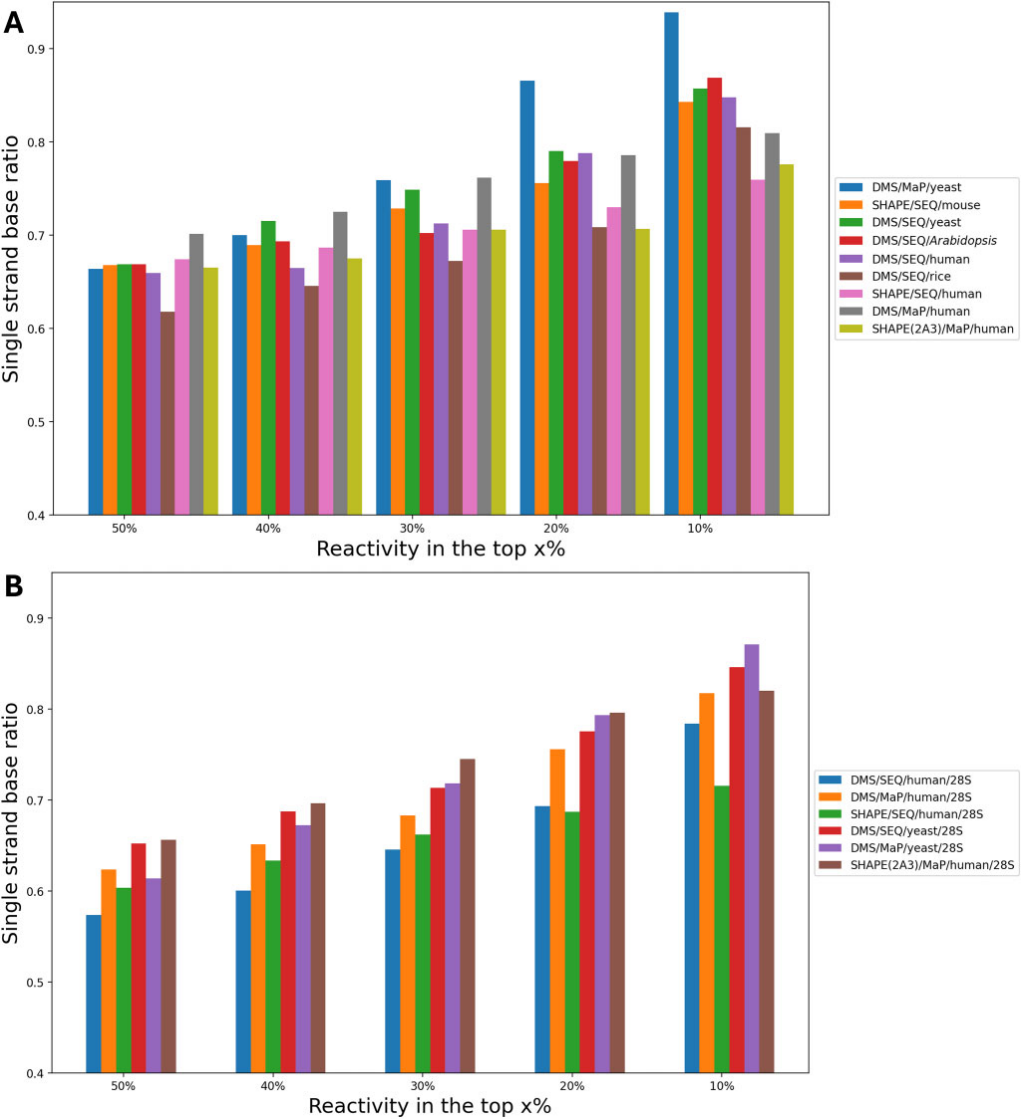
Accuracy of base pair identification by reactivity range. RNA chemical probing data were mapped to 18S/25S/28S rRNA, and the percentage of unpaired bases is shown for the top reactive bases. The horizontal axis shows the top *x*% of reactivity in the RNA molecule. The vertical axis shows the percentage of top *x*% reactive bases that are not involved in canonical base pairing. Panel (**A**) shows the results for 18S rRNA. Panel (**B**) shows the results for 25S/28S rRNA.

High reactivity indicates an unpaired RNA structure. It is important to know the threshold of reactivity that correlates with structure. Therefore, we investigated whether bases with the top *x*% reactivity in an RNA molecule match their structure (Figure 5). The structure data of 18S/25S/28S rRNA for each species were downloaded from RNAcentral (Tables 1 and 2). Bases with higher reactivity within a molecule consistently showed higher accuracy in reflecting the secondary structure (Figure 5). The accuracy of predicting bases not involved in Watson–Crick pairs was 72–94% for those with the top 10% reactivity in 18S/28S rRNA data. It has been reported that SHAPE data with high reactivity are useful for structure prediction (48,49), and this trend was found to be consistent across all transcriptome-scale experiments, including DMS experiments. In general, secondary structures within cells are dynamic and undergo constant changes (50), and considering that the modification reaction by the probe is more likely to occur when the bases are unpaired, dynamic RNA structural changes are expected to reduce accuracy. Therefore, the present results could be considered as sufficiently accurate.

In previous studies, reactivity has been used for free-energy parameter adjustment, in which an adjustment of ln(1 + reactivity) is applied (14). This means that when reactivity is high, a large adjustment is applied to free energy. From the analysis of the data in this study, a limited number of bases showed high reactivity, while most of the others had a reactivity of ∼0. It was also clear that these bases with high reactivity were highly correlated with unpaired structures. Therefore, we believe that the adjustment method that only adjusts for bases with high reactivity is a reasonable adjustment method for RNA chemical probing data at the transcriptome scale in eukaryotes. Kawaguchi *et al.* proposed a method using reactivity with high reproducibility between replicates (51). In the present study, we also found that some bases with high reactivity have high reproducibility between replicates (data not shown). Therefore, we believe that using reproducibility of reactivity is a reasonable approach.

### Relationship between reactivity and base pair probability

In a Kaggle competition for predicting RNA reactivity from sequence, accuracy was improved by inputting base pair probabilities calculated from sequence information, and this approach won the competition (52). Therefore, it can be considered that base pair probabilities hold (at least part of) the reactivity information. We investigated the relationship between base pair probability and reactivity by calculating base pair probabilities from 18S rRNA sequences. Supplementary Figure S3 shows the percentage of bases with base pair probabilities of ≥0.5 that were included in the bases showing the top *x*% reactivity on the horizontal axis. Bases with high base pair probabilities are expected to be in probe-inaccessible structures, and therefore low reactivity is expected. Supplementary Figure S3 shows a downward trend; i.e. the number of bases with high base pair probabilities decreased for bases with higher reactivity. However, there did not seem to be a consistent trend, as in Figure 5. We propose two main reasons for this weaker correlation between base pair probability and reactivity compared to the RNA structures in RNAcentral. First, RNA secondary structure prediction is known to be inaccurate for long RNAs (>500 bases) (53). Second, rRNA is stabilized energetically during biosynthesis by the support of many molecules including chaperones (54,55), and the structures registered in RNAcentral are based on information from RiboVision (56), which considers various *in vivo* environments. In contrast, the base pair probabilities were calculated only from sequence information.

Regions with concordant secondary structure predictions from RNAcentral and RNAfold are anticipated to exhibit high base pairing probability and low reactivity. However, as shown in Table 4, certain bases displayed high reactivity despite these predictions. We investigated whether the bases with high reactivity, despite being predicted to form secondary structures in RNAcentral and having high base pair probabilities, formed base pairs in the actual 3D structure. Reactivity was determined using mouse SHAPE/SEQ data, and 3D structures were based on PDB ID: 7CPU. According to RNAcentral, 887U forms a wobble base pair with 903G (Supplementary Figure S4A). However, in the 3D structure of PDB ID: 7CPU, 887U corresponds to 886U and is not involved in hydrogen bonding (Supplementary Figure S4B). Additionally, RNAcentral predicted that 1762G forms a Watson–Crick base pair with 1774C (Supplementary Figure S4C). In contrast, in the 3D structure of PDB ID: 7CPU, 1762G corresponds to 1761G and is not involved in base pair formation (Supplementary Figure S4D). Furthermore, the region around 1762G is predicted to form a stem structure by RNAcentral, but no hydrogen bonds are formed to maintain this stem structure in the 3D structure. These results support the hypothesis that high reactivity indicates an unpaired structure and suggest that bases with the top 10% of reactivity are a reliable signal of an unpaired structure in the biochemical environment.

**Table 4.** Bases with a sum of base pair probability (RNAfold) of ≥0.995 and reactivity within the top 10%

Base position	Base	Base pair probability	Reactivity	RNAcentral	3D structure
772	U	0.997	0.05	Not paired	Not solved
773	A	0.999	0.06	Not paired	Not solved
774	G	0.999	0.13	Not paired	Not solved
775	C	0.999	0.56	Not paired	Not solved
872	A	0.996	0.06	Not paired	Not paired
887	U	0.999	0.05	Paired	Not paired
888	A	0.98	0.09	Not paired	Not paired
1762	G	0.999	0.04	Paired	Not paired

The table summarizies the RNAcentral secondary and 3D structures of bases with a marginalized base pair probability (RNAfold) of ≥0.995 and reactivity within the top 10%. Reactivity was determined using mouse SHAPE/SEQ data, and 3D structures were based on a mouse ribosomal structure (PDB ID: 7CPU). In RNAcentral, paired bases are indicated as ‘Paired’ and unpaired bases are indicated as ‘Not paired’. For 3D structures, some bases lack resolved molecular models and are indicated as ‘Not solved’. Unpaired bases are also indicated as ‘Not paired’.

### Relationship between reactivity and solvent accessibility of 3D structures

**Figure 6.**
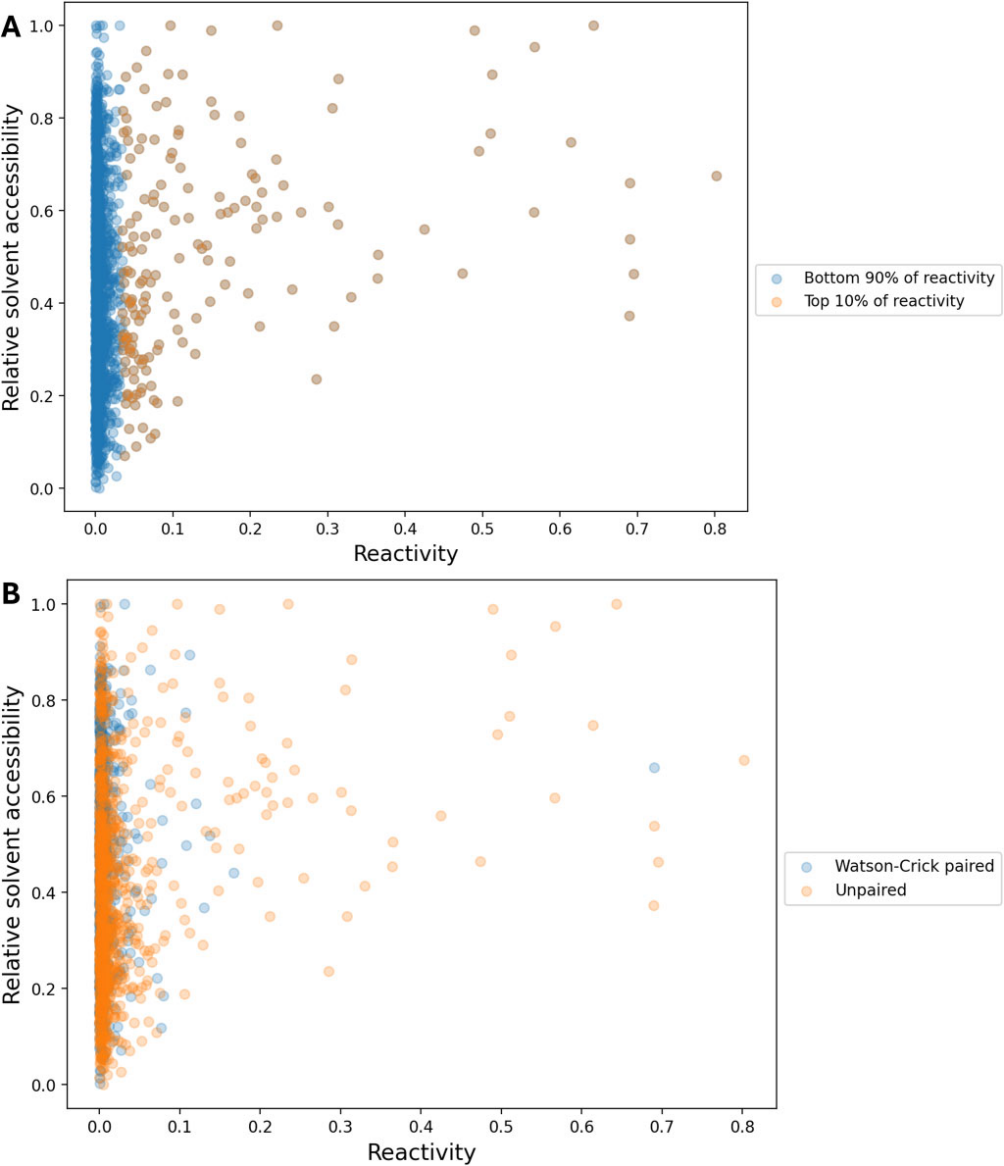
Relationship between solvent accessibility and reactivity. The horizontal axis shows the reactivity of mouse 18S rRNA from mouse cell SHAPE-seq data (10). The vertical axis shows the relative solvent accessibility calculated from the ribosome small subunit (PDB ID: 7CPU) of the mouse ribosomal structure. The panel (**A**) shows the relationship for the bases with the top 10% reactivity and the bases with the bottom 90% reactivity. The panel (**B**) shows that the relationship for the unpaired bases (not forming canonical base pairs) and the bases forming canonical base pairs. Since the reactivity distribution is peak at zero, the plots near zero overlap. Both Watson–Crick paired and unpaired plots are included in the region around zero. Refer to Supplementary Figure S1C for the distribution of reactivity of these data.

Reactivity in RNA chemical probing experiments is affected not only by the secondary structure of RNA but also by its tertiary structure. There are many reports on the relationship between the solvent accessibility and reactivity of RNA structures (18). As shown in the present study, a large portion of the reactivity of RNA chemical probing experiments was close to zero (Figure 1). Therefore, it is important to establish criteria for determining which reactivity values are related to solvent accessibility. The 3D structures of several eukaryotic ribosomes have been solved, and we investigated the relationship between 18S rRNA solvent accessibility and reactivity. The 3D structures of the mouse (PDB ID: 7CPU) (43), yeast (PDB ID: 8CCS) (44) and human (PDB ID: 8JDK) (45) ribosomes were downloaded from PDB and the solvent accessibility of each ribosomal small subunit was calculated (see the ‘Materials and methods’ section). The relationship between the solvent accessibility and reactivity of these structures is shown in Figure 6 and Supplementary Figure S5. Based on the secondary structure analysis results shown in Figure 5, the top 10% of the data had high secondary structure prediction accuracy, so we also used the top 10% data for this analysis. The blue plots in Figure 6 and Supplementary Figure S5 represent the data with the bottom 90% reactivity, while the orange plots represent the data with the top 10% reactivity. For the mouse, when the data were limited to the top 10%, the correlation coefficient was 0.32, showing a weak correlation (Figure 6), whereas the correlation coefficient of all data was 0.15, suggesting that the correlation coefficient was lowered in the bottom 90% (Figure 6). For the other data (Supplementary Figure S5), there was a tendency for the top 10% of reactive bases not to have low solvent accessibility.

Chemical probes are reported to be biased toward structures that are easy to react with (19,21,23,25,38), which is consistent with the observation that even bases that are exposed to solvent do not have increased reactivity (Figure 6A). There were many cases in which bases that were not base-paired and had high solvent accessibility did not have high reactivity (Figure 6B), suggesting that difficult-to-react bases may not be reflected in reactivity even if they are exposed to solvent.

Some studies have only considered solvent-accessible bases when evaluating chemical probing data using rRNAs with known 3D structures (8,42). This is reasonable given the experimental limitations that probing reagents only react with regions that are exposed to solvent, as ribosomes are huge and complex structures. However, there are also many RNAs with unknown structures, so it is necessary to consider all bases for the evaluation of reactivity thresholds, as in the present study. As can be seen from the results shown in Figure 6 and Supplementary Figure S6, while high reactivity tends to be associated with high solvent accessibility, low reactivity does not always represent low solvent accessibility. Additionally, while high reactivity suggests a low probability of base pairing (Figure 5 and Supplementary Figure S3), low reactivity does not always indicate base pairing. This was consistent across transcriptome-scale RNA chemical probing experiments in mouse, human and yeast. These are important points to consider when interpreting RNA chemical probing data.

## Conclusions

In this study, we analyzed the results of RNA chemical probing experiments performed at the transcriptome scale in eukaryotes. While RNA chemical probing data have been shown to be an effective signal, especially for the top 10% reactivity data, for detecting base-pairing states within RNA structures, the moderate overlap between the distribution of reactivity and the background distribution suggests that it would be currently difficult to completely distinguish and detect the presence or absence of base pairs based solely on the reactivity values. Recently, improvements in probing methods have been made, and it has also been confirmed that a new experimental approach using the 2A3 reagent can detect base pairs more accurately. In the coming era of data science, to overcome the difficulty of detecting RNA structures solely from reactivity values of RNA chemical probing, it will be necessary to combine new SHAPE experiments, such as those using the 2A3 reagent, with machine learning techniques such as those used in Kaggle, to achieve more accurate detection of RNA structures.

## Supplementary Material

lqaf001_Supplemental_File

## Data Availability

The data underlying this article are available in SRA at https://www.ncbi.nlm.nih.gov/sra, and can be accessed under accession numbers SRR815612, SRR815617, SRR1057942, SRR1058055, SRR933552, SRR933551, SRR5297223, SRR5297221, SRR3929621, SRR3929626, SRR3929628, SRR3929619, SRR3929620, SRR7618815, SRR7618817, SRR7618833, SRR7618835, SRR12235552 and SRR12235545.
