# Supplementary material for: Consistent features observed in structural probing data of eukaryotic RNAs
Source: NAR Genom Bioinform. 2025 Jan 30;7(1):lqaf001. doi: 10.1093/nargab/lqaf001 (PMC11780854; doi:10.1093/nargab/lqaf001)
Supplement: lqaf001_Supplemental_File [file lqaf001_supplemental_file.docx]

**Supplementary data**

**Title**

Consistent features observed in structural probing data of eukaryotic RNAs

Figure S1

A


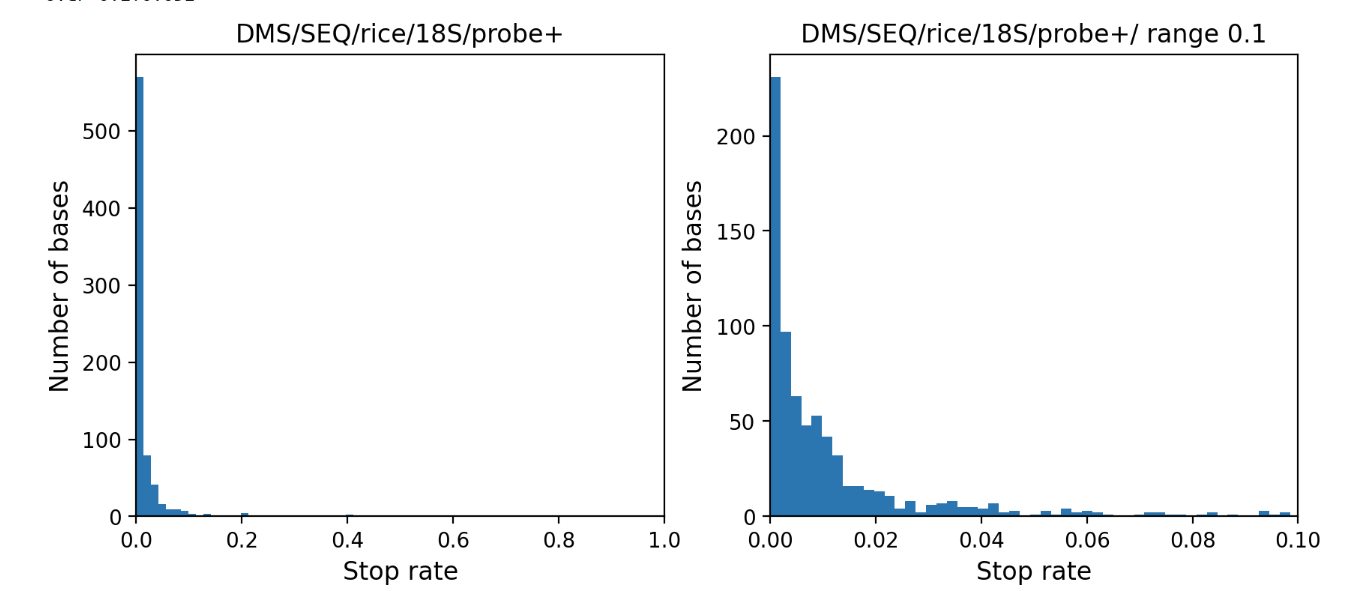


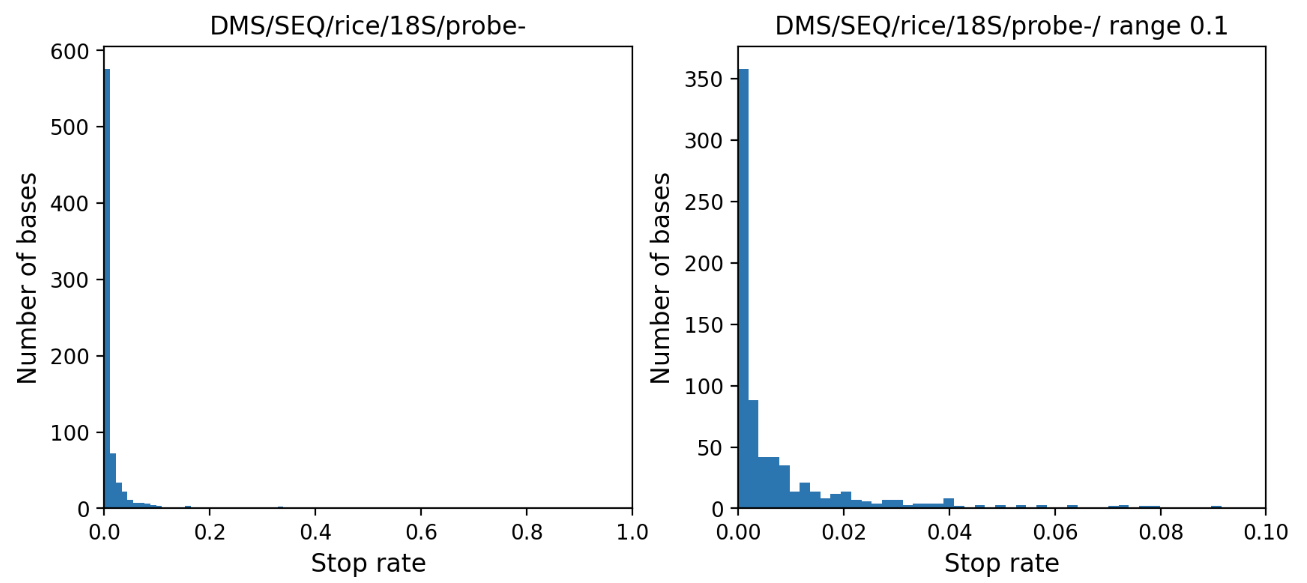


B


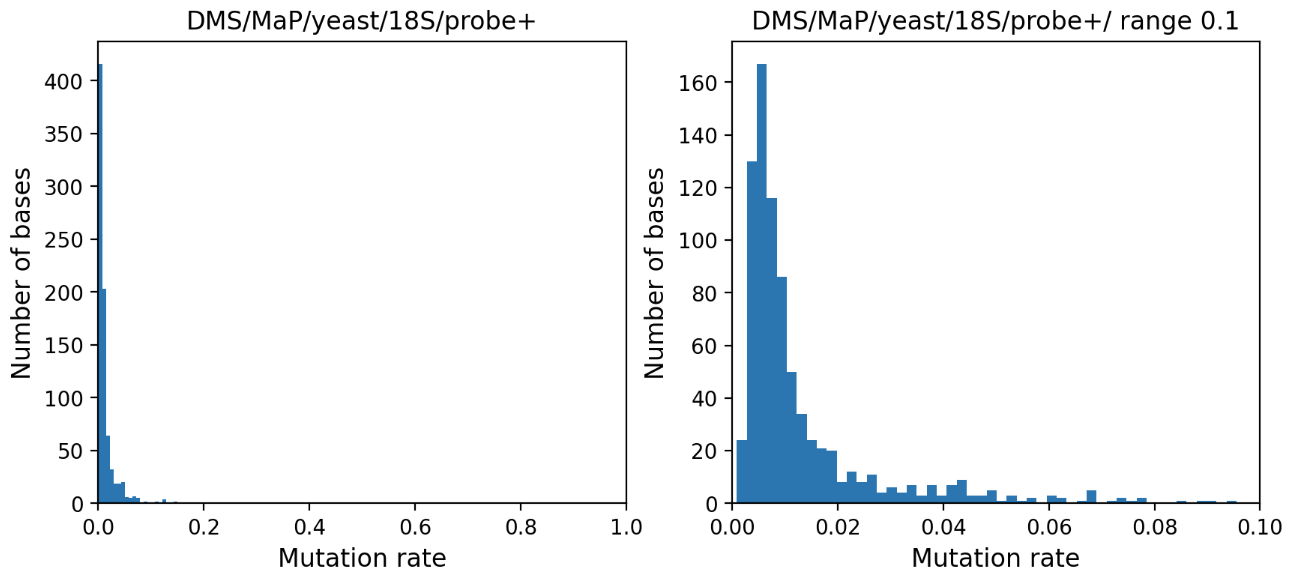


C


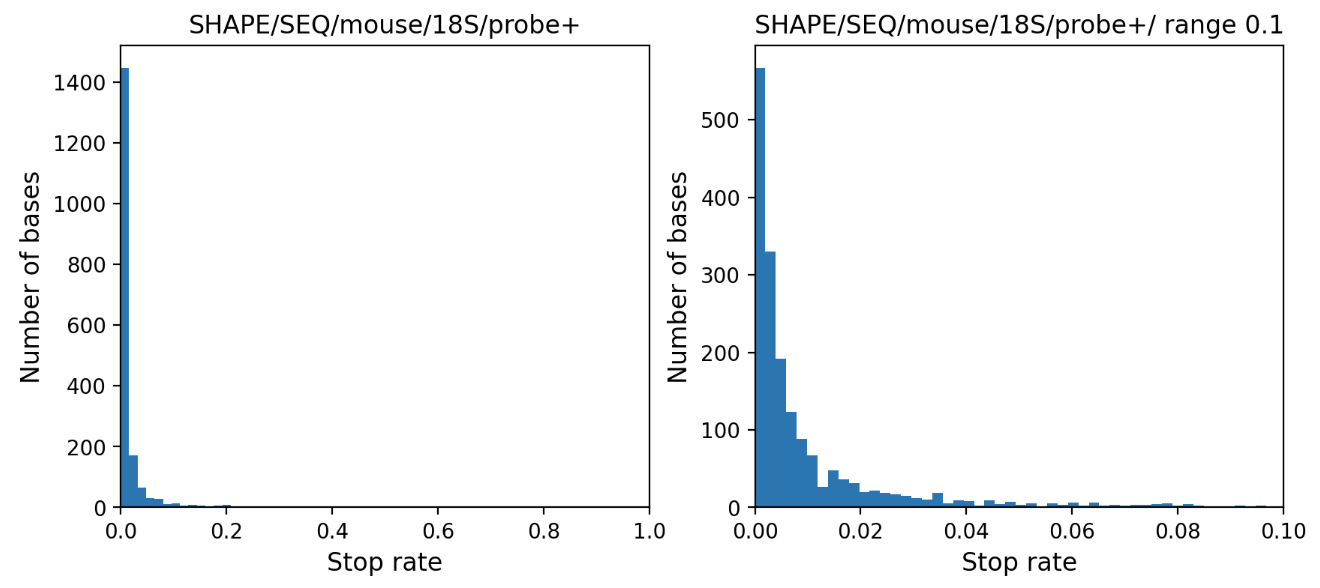


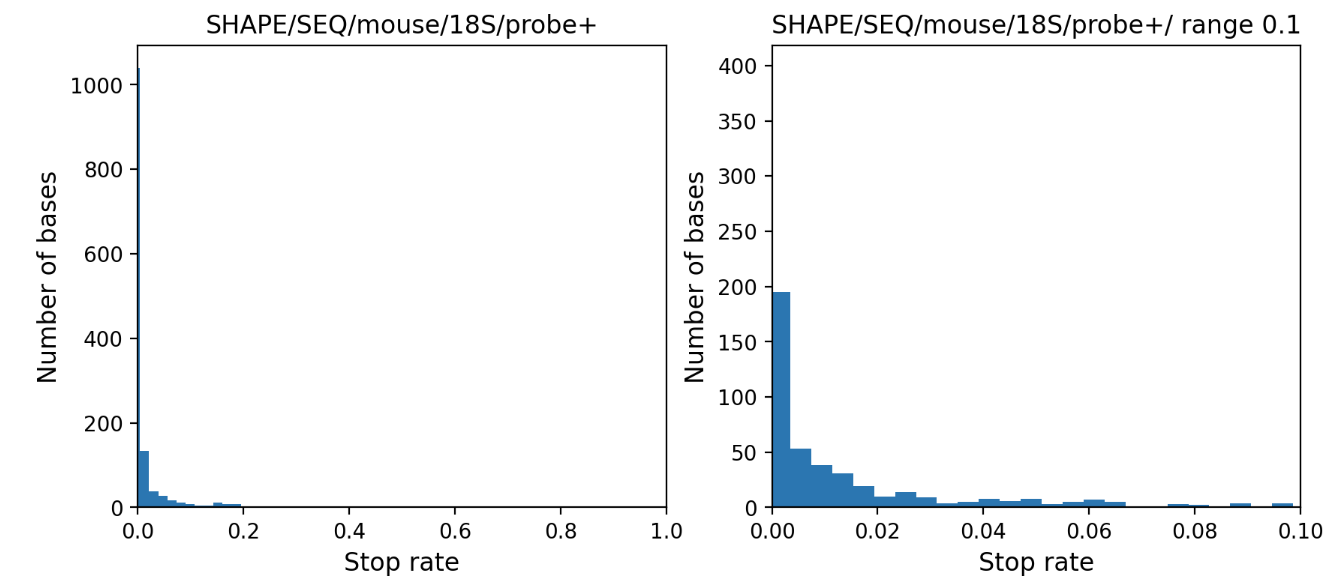


D


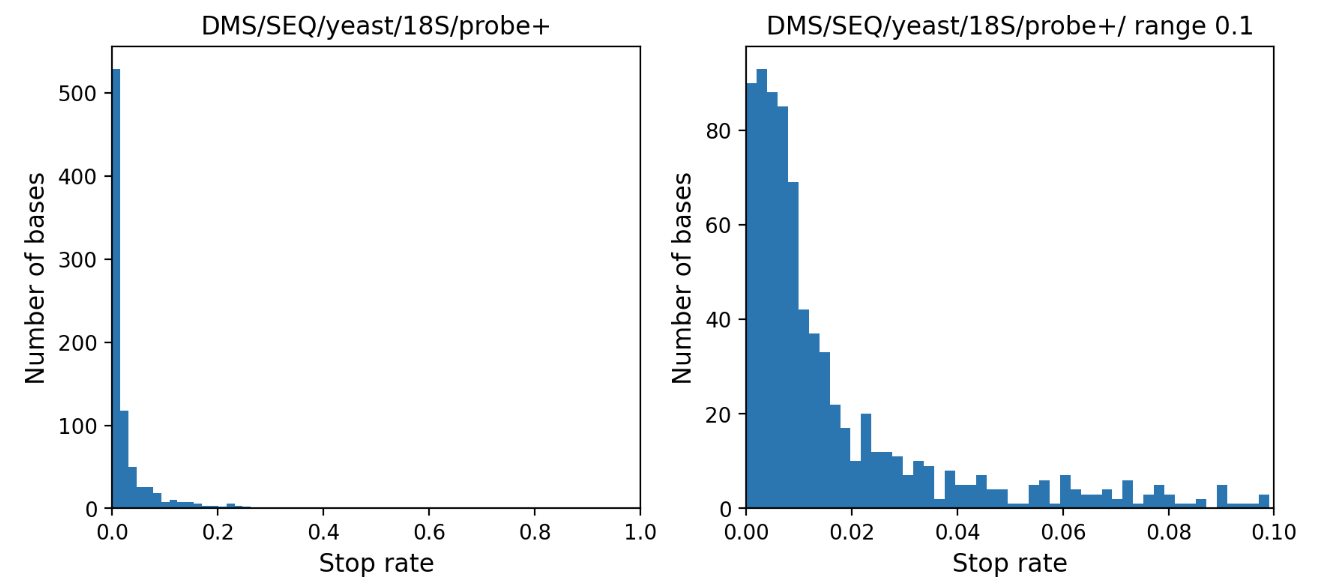


E


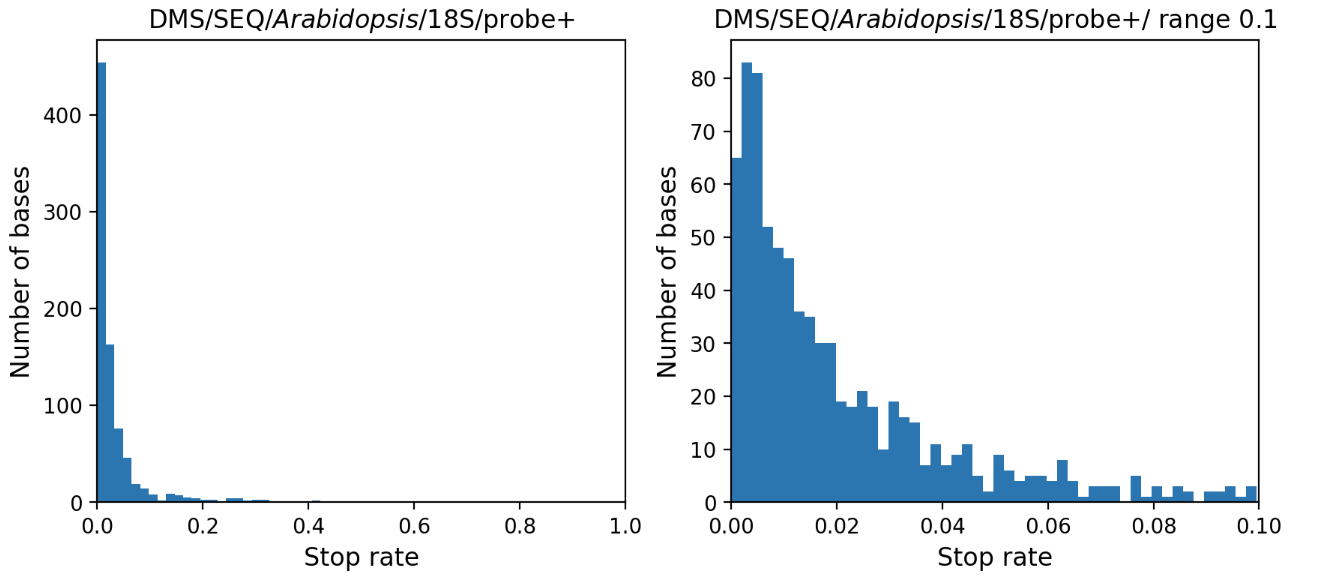


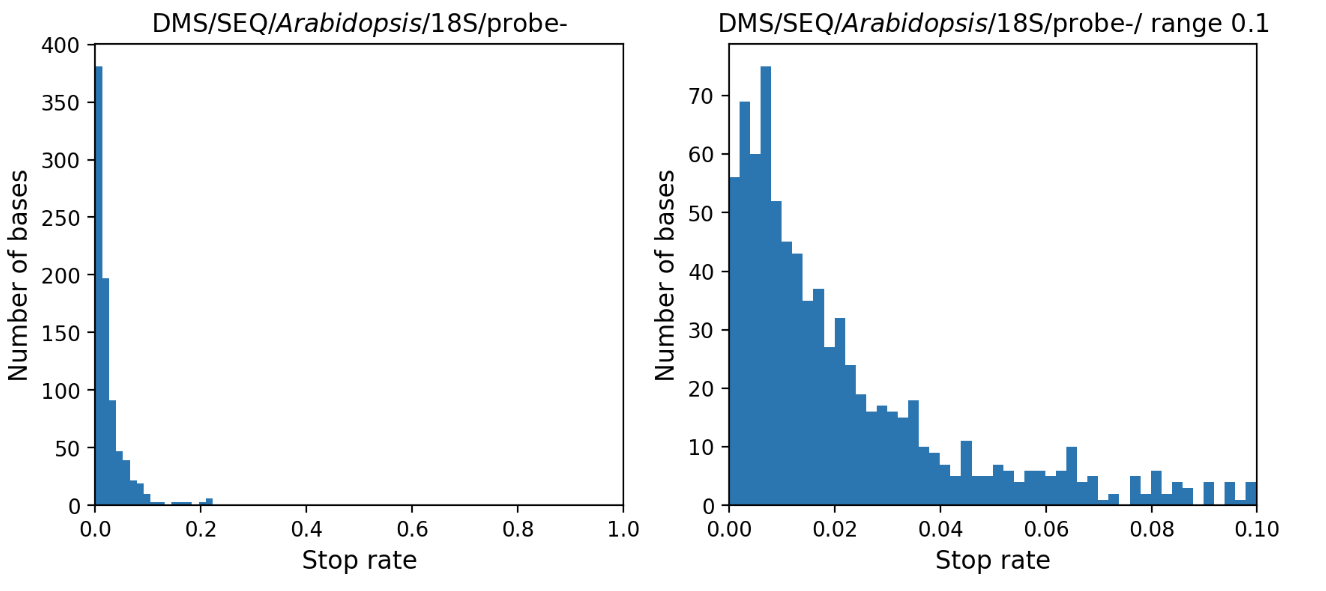


F


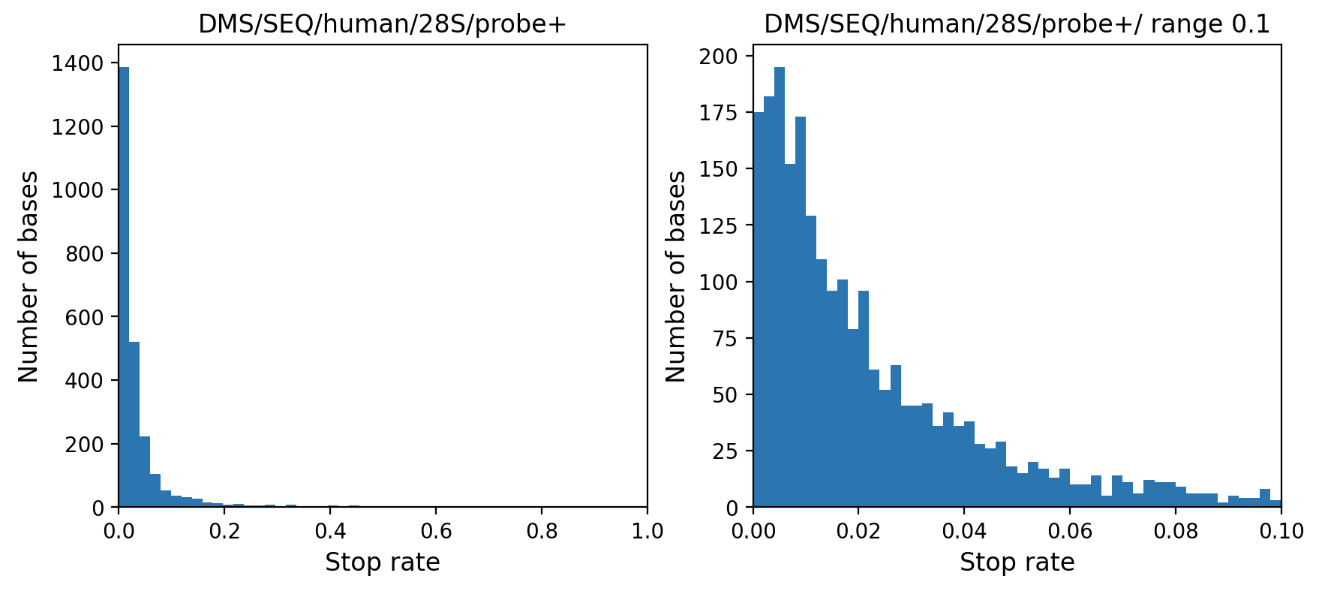


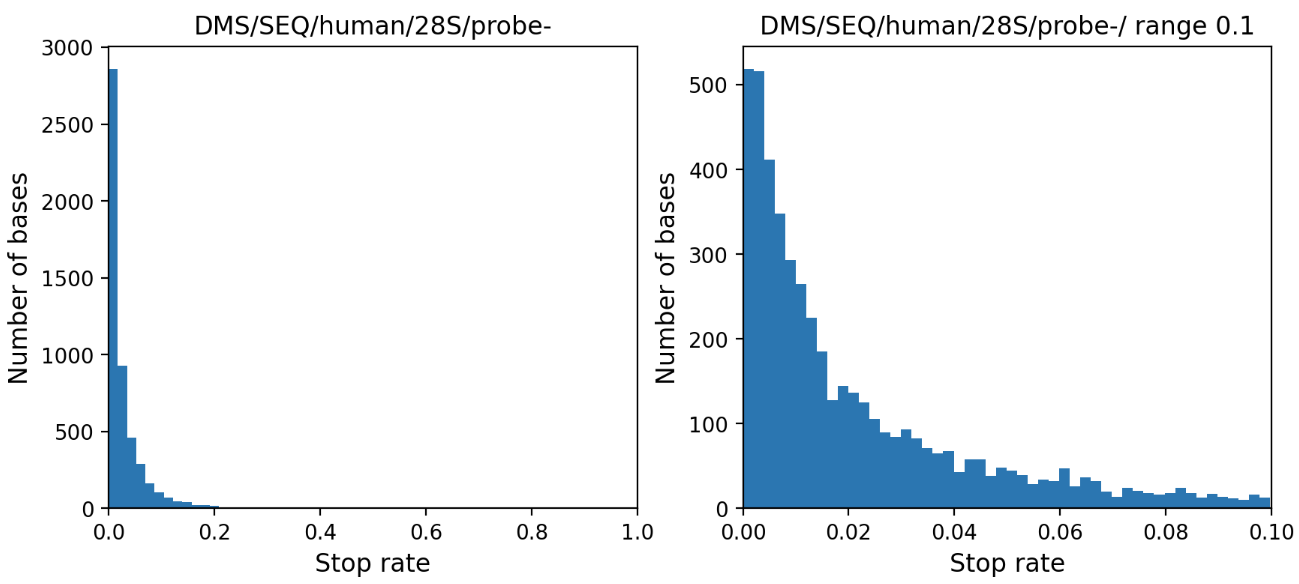


G


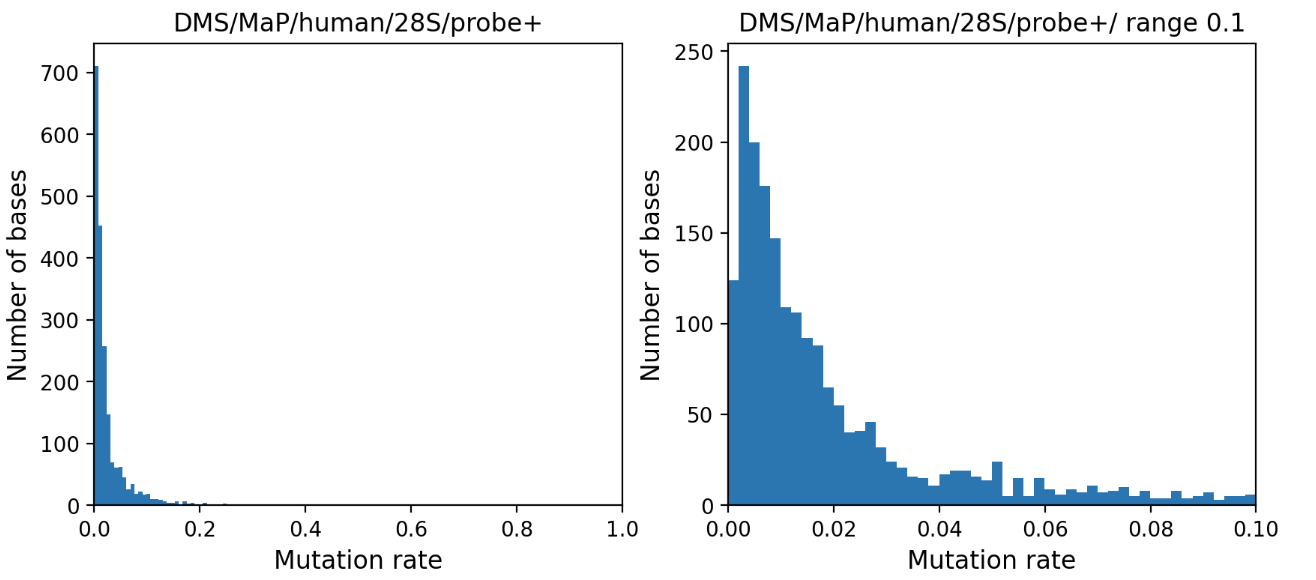


H


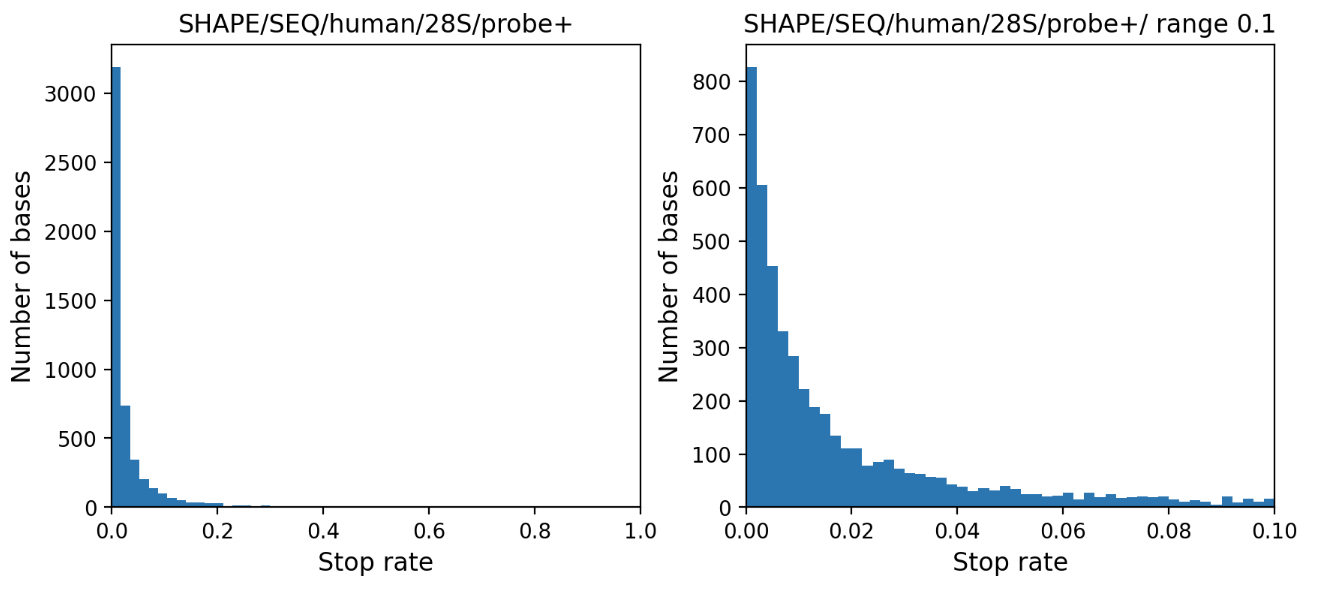


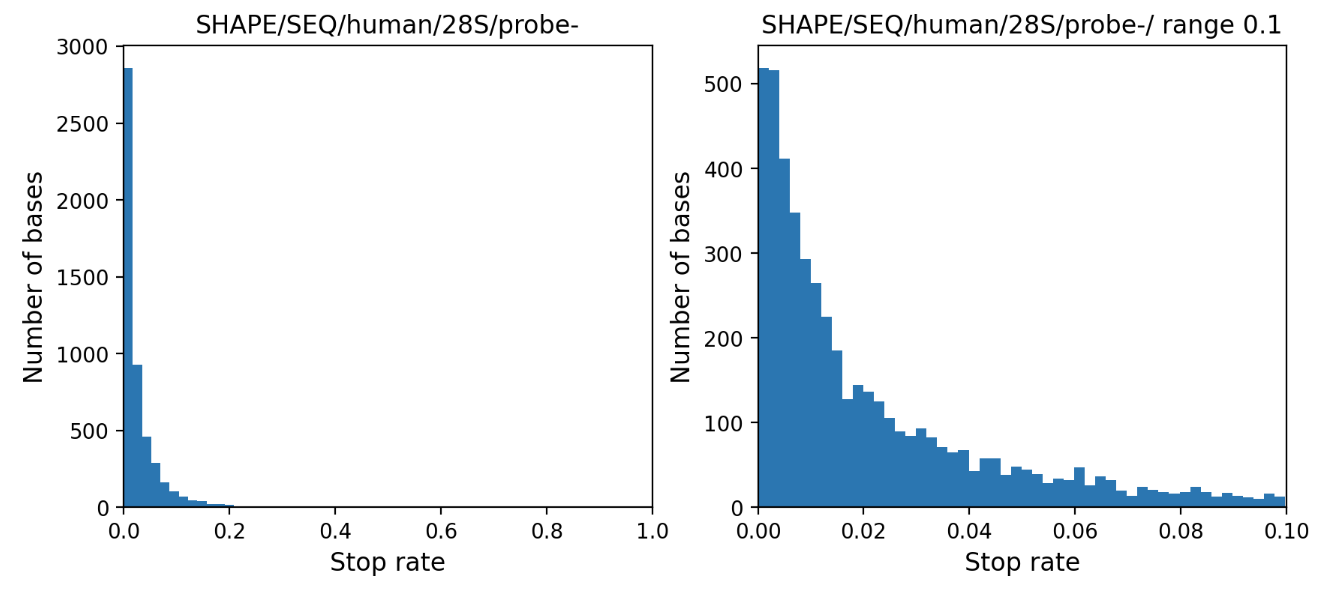


I


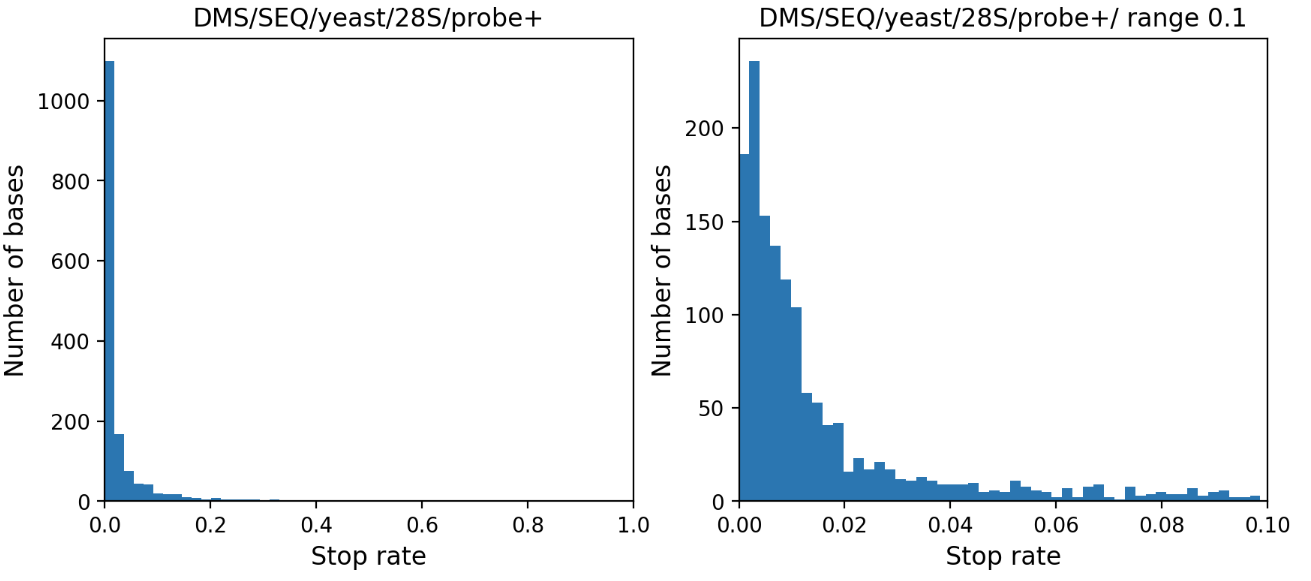


J


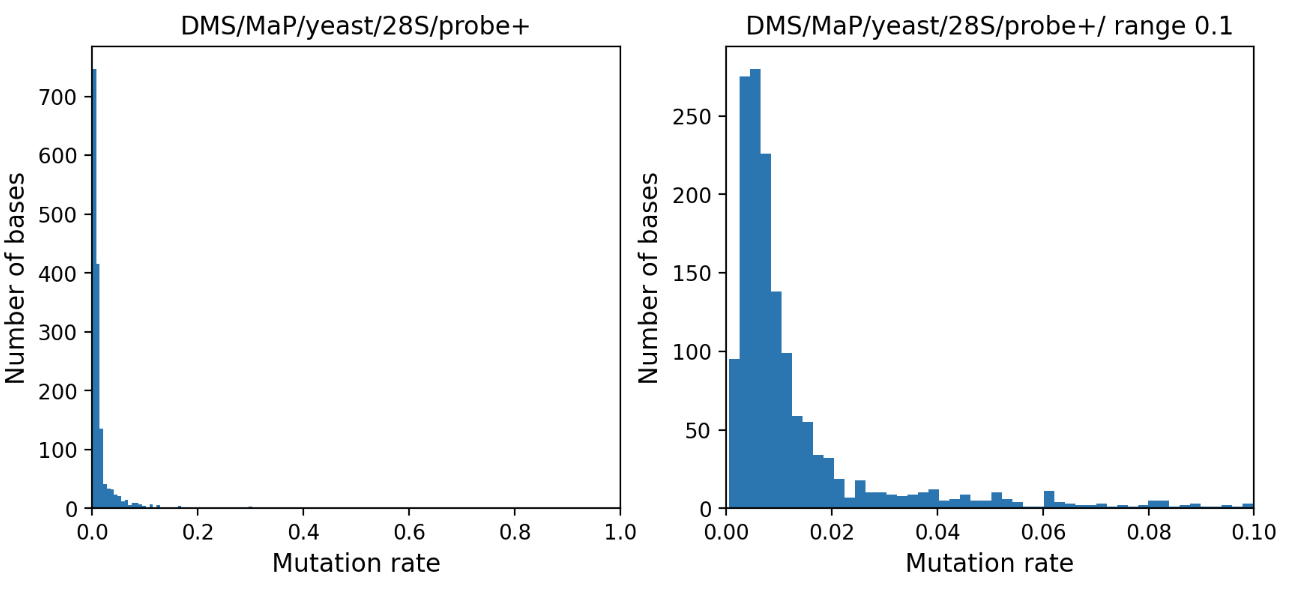


K


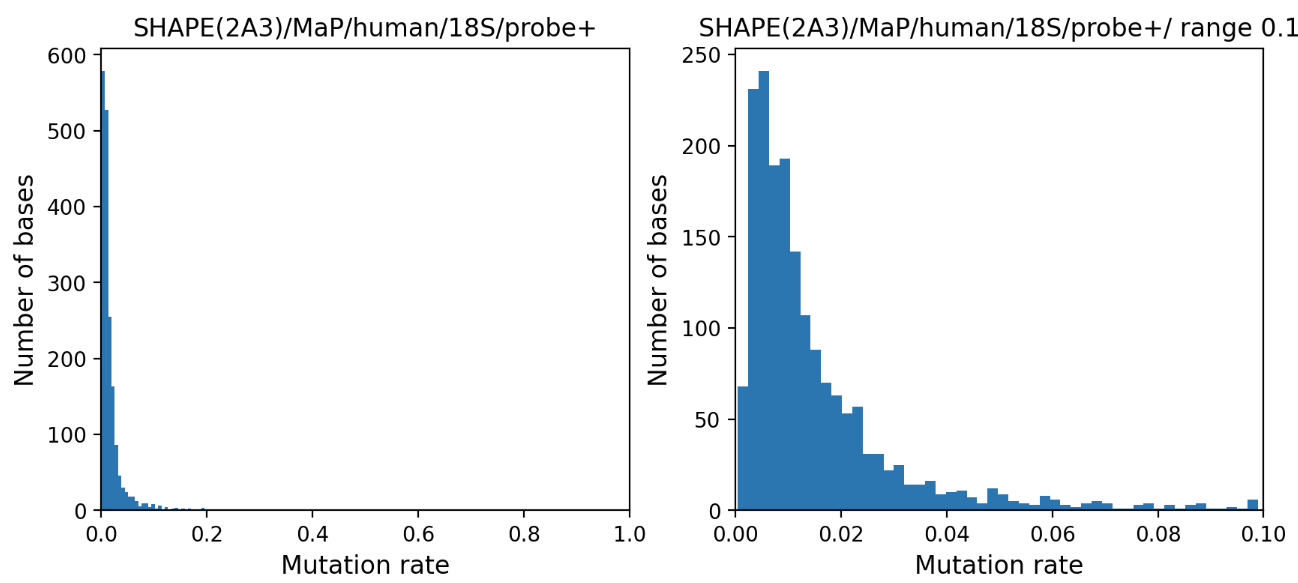


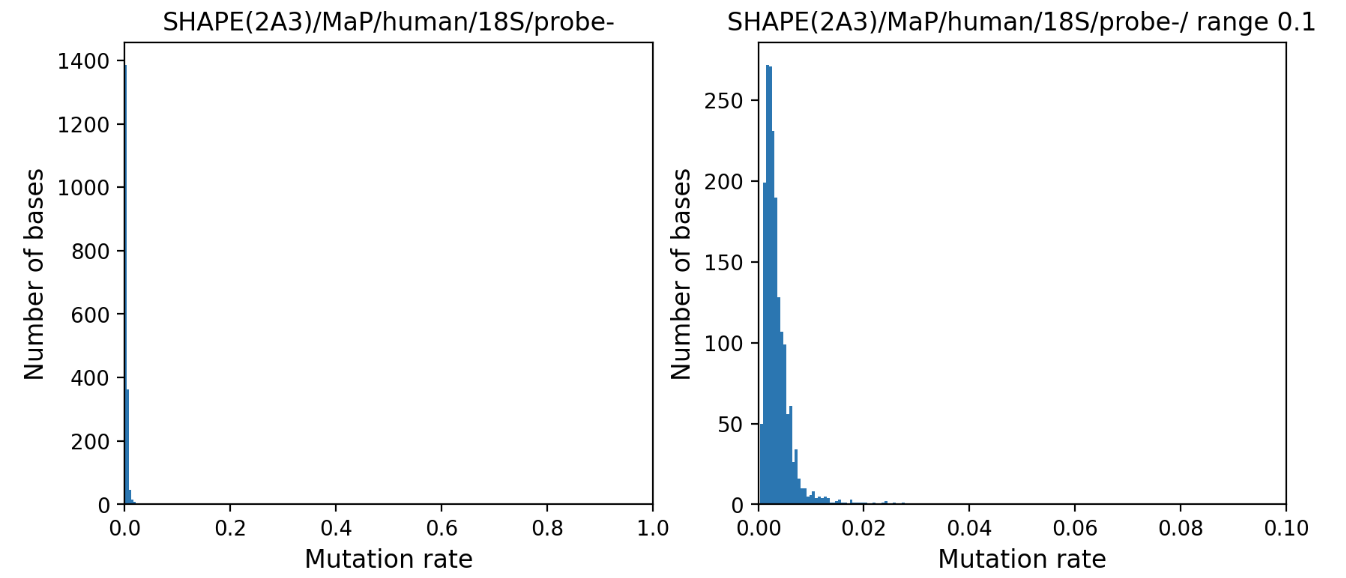


L


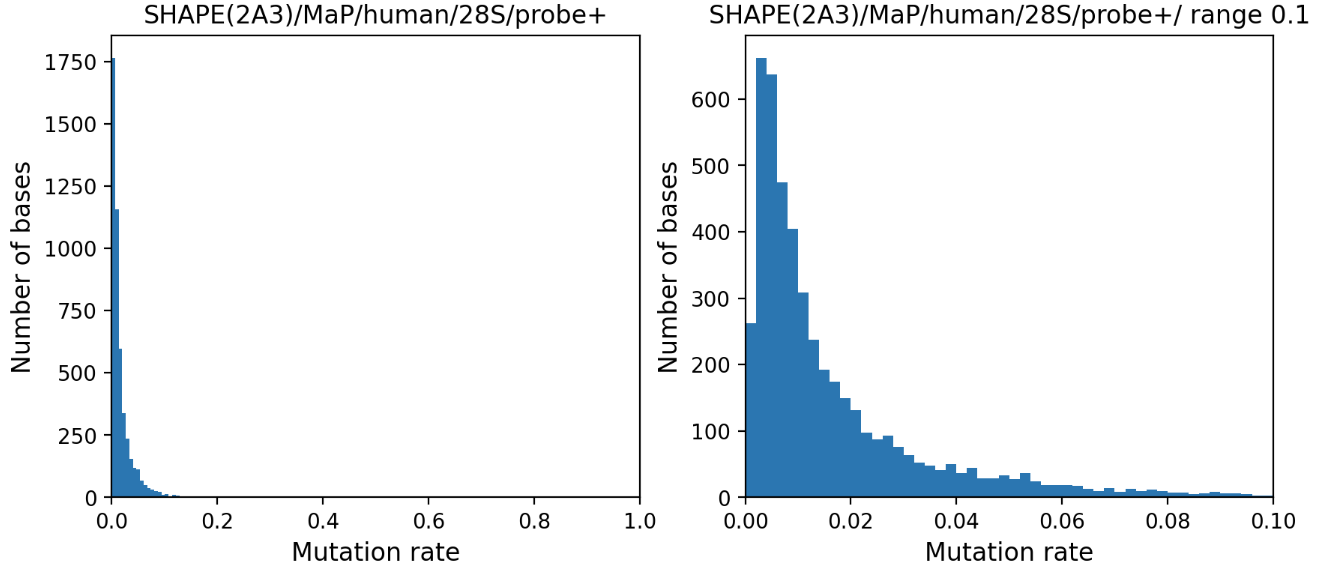


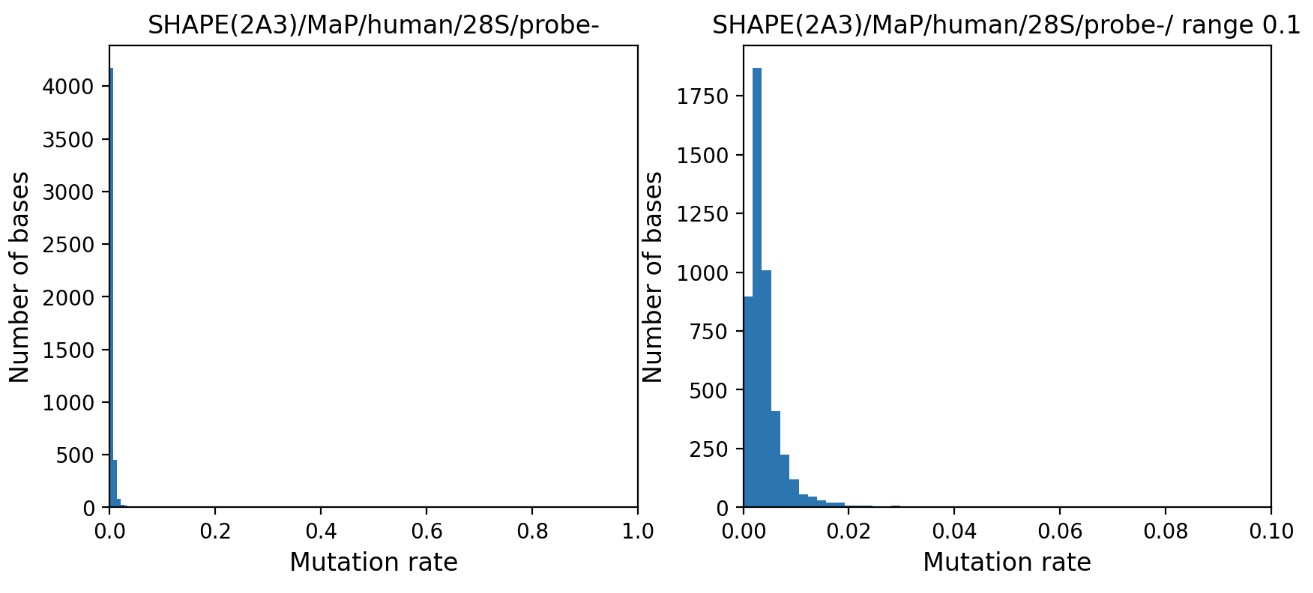


M


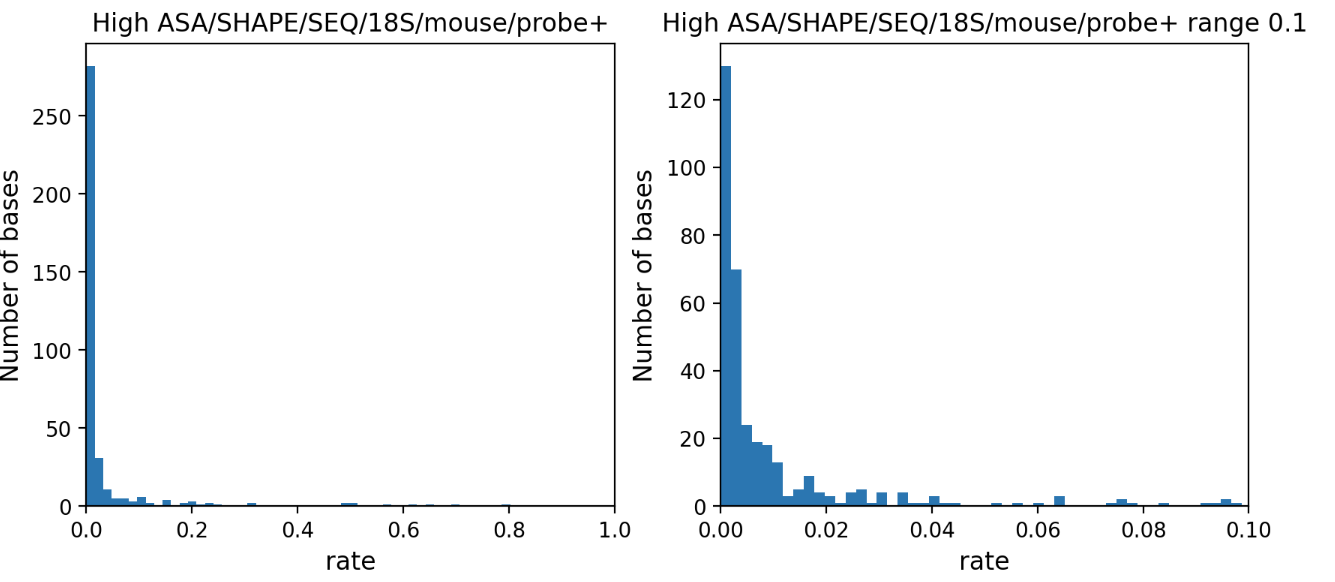


N


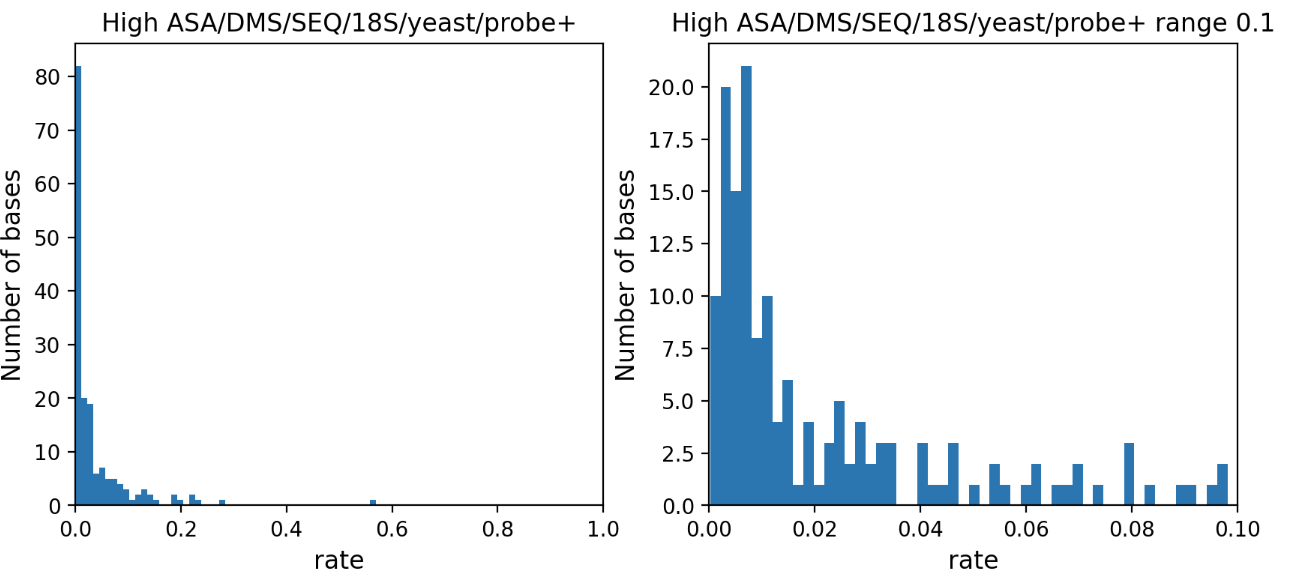


O


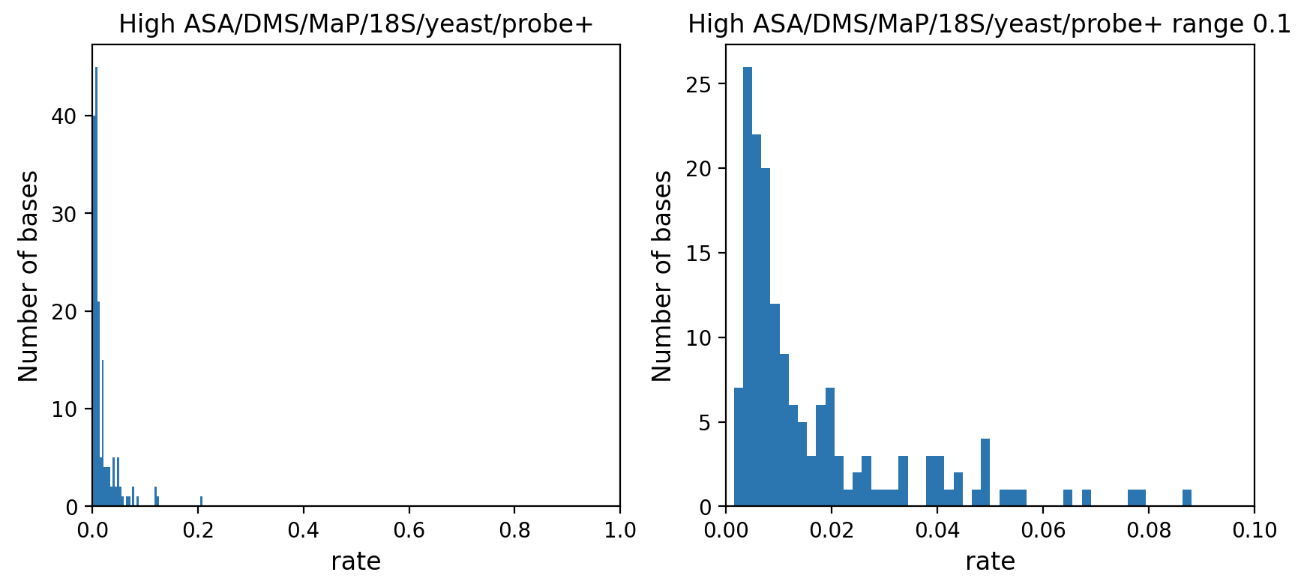


P


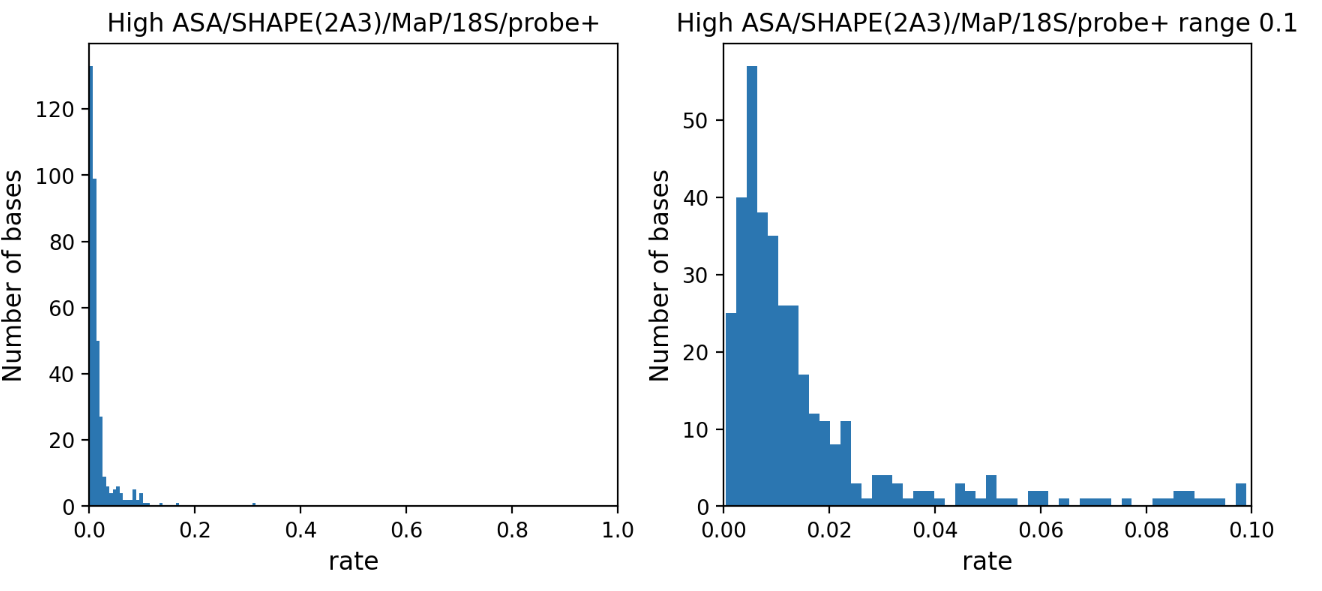


Q


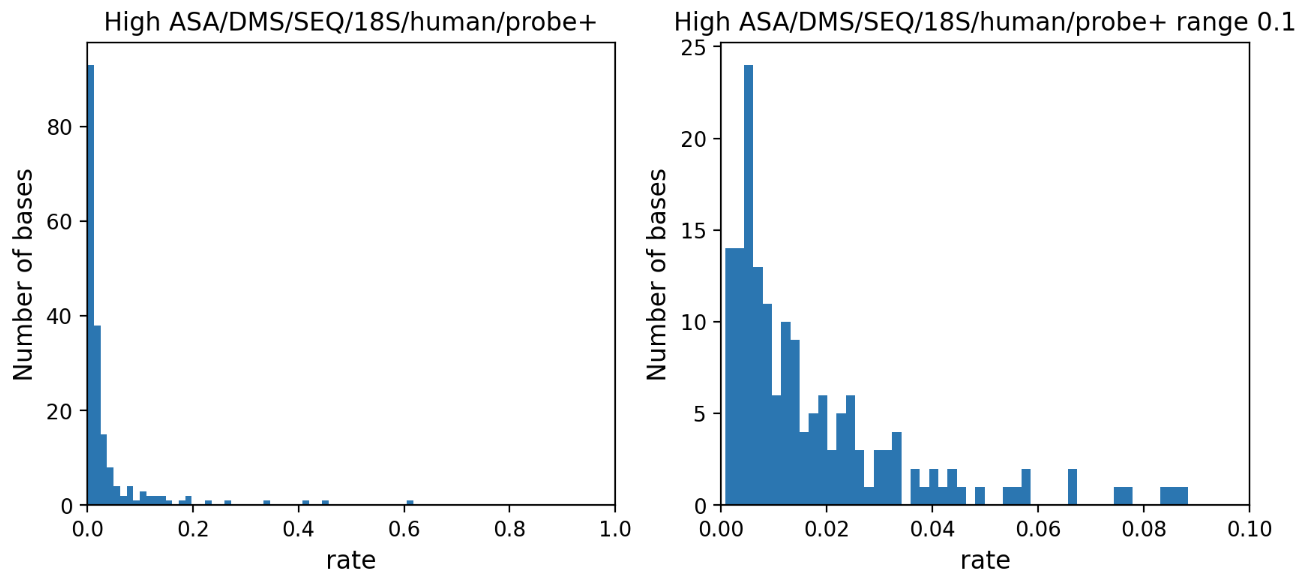


R


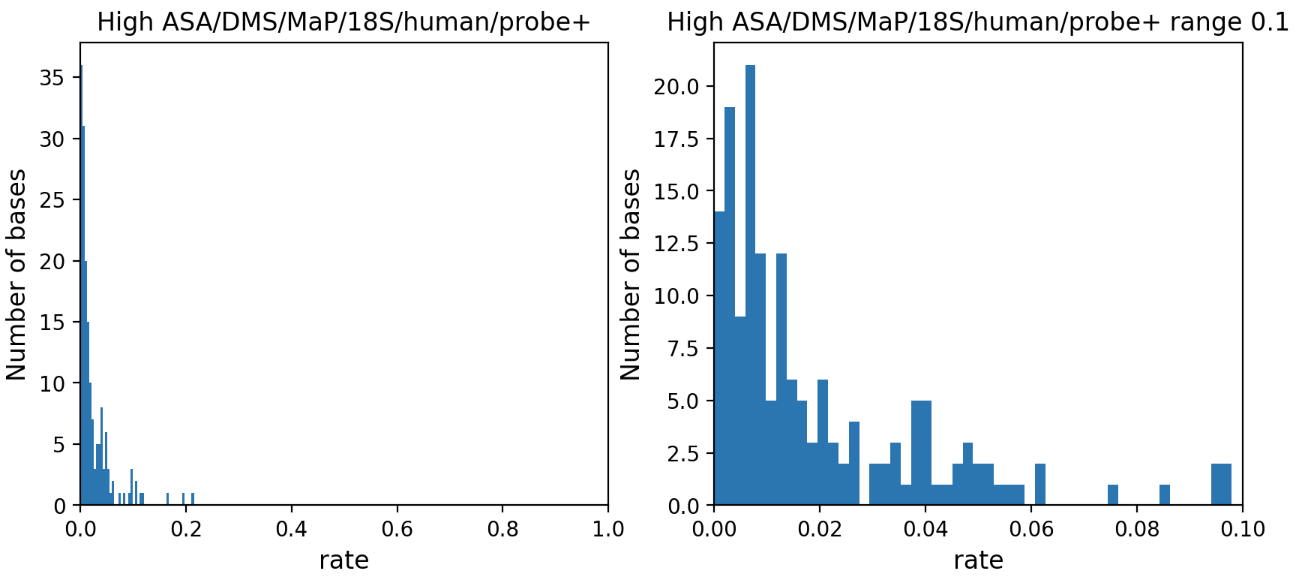


S


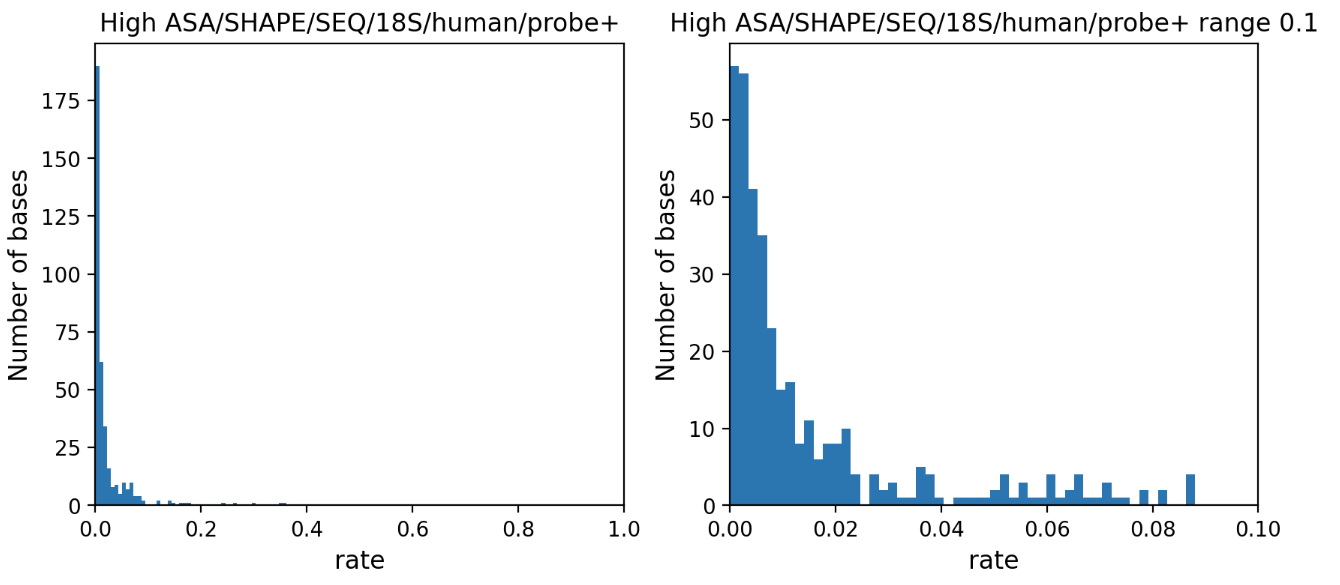


Figure S1. Histograms of the stop/mutation rate of RNA chemical probing experiments

The read data of RNA chemical probing experiments were mapped to 18S/25S/28S rRNA, and the reverse transcription stop rate and mutation rate were calculated. The horizontal axis represents the stop or mutation rate, while the vertical axis indicates the number of bases associated with the stop or mutation rate. The left-side panel displays a histogram in the range of 0 to 1.0, while the right-side panel is limited to the range of 0 to 0.1 for the same histogram. The bottom panels show the histogram of stop rates for control experiments (without DMS/SHAPE reagents), if available. The mutation rate was calculated by dividing the number of specific bases with mutations by the total number of times that base was read. Similarly, the stop rate was calculated by dividing the number of specific bases at which reverse transcription was halted by the total number of times that base was read. (**A**) Rice DMS reverse transcription stop profile mapped to 18S. (**B**) Yeast DMS mutational profile mapped to 18S. (**C**) Mouse SHAPE reverse transcription stop profile mapped to 18S. (**D**) Yeast DMS reverse transcription stop profile mapped to 18S. (**E**) *Arabidopsis* DMS reverse transcription stop profile mapped to 18S. (F) Human DMS reverse transcription stop profile mapped to 28S. (G) Human DMS mutational profile mapped to 28S. (H) Human SHAPE reverse transcription stop profile mapped to 28S. (I) Yeast DMS reverse transcription stop profile mapped to 25S. (J) Yeast DMS mutational profile mapped to 28S. (K) Human SHAPE (2A3) mutational profile mapped to 18S. (L) Human SHAPE (2A3) mutational profile mapped to 28S. (M) Mouse SHAPE stop profile mapped to 18S restricted to bases with top 20% of solvent exposure. (N) Yeast DMS stop profile mapped to 18S restricted to bases with top 20% of solvent exposure. (O) Yeast DMS mutational profile mapped to 18S restricted to bases with top 20% of solvent exposure. (P) Human SHAPE (2A3) mutational profile mapped to 18S restricted to bases with top 20% of solvent exposure. (Q) Data from human DMS stop profiles restricted to bases with top 20% of solvent exposure. (R) Data from human DMS mutation profiles restricted to bases with top 20% of solvent exposure. (S) Data from human DMS stop profiles restricted to bases with top 20% of solvent exposure.

Figure S2

A. DMS/SEQ/rice


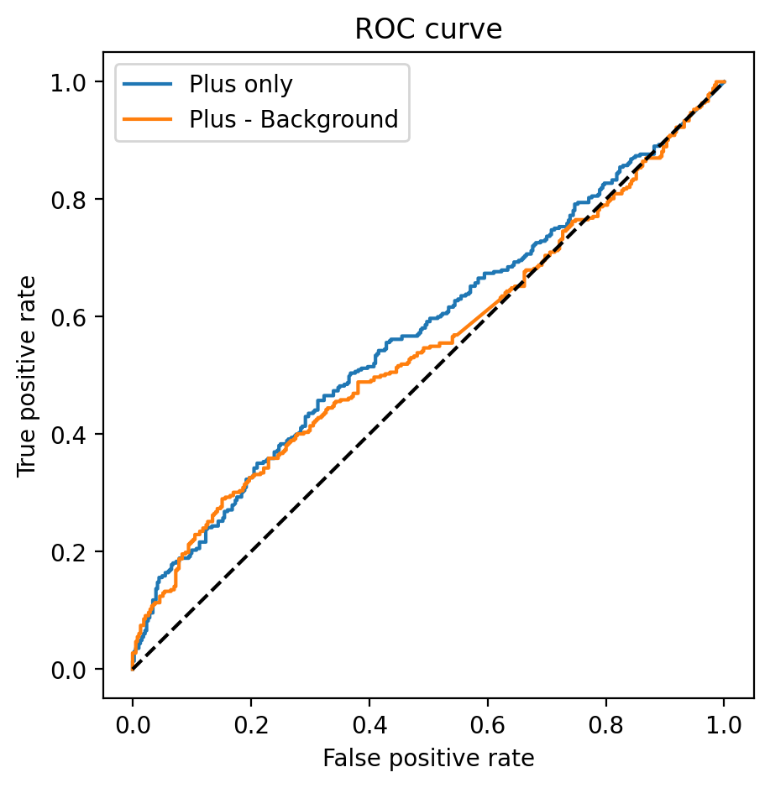


B. SHAPE/SEQ/mouse


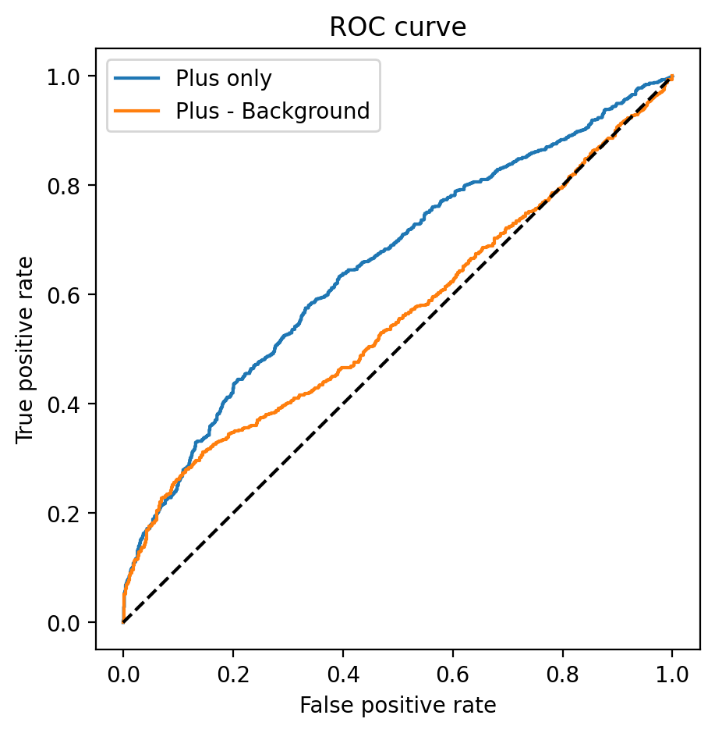


C. DMS/SEQ/yeast


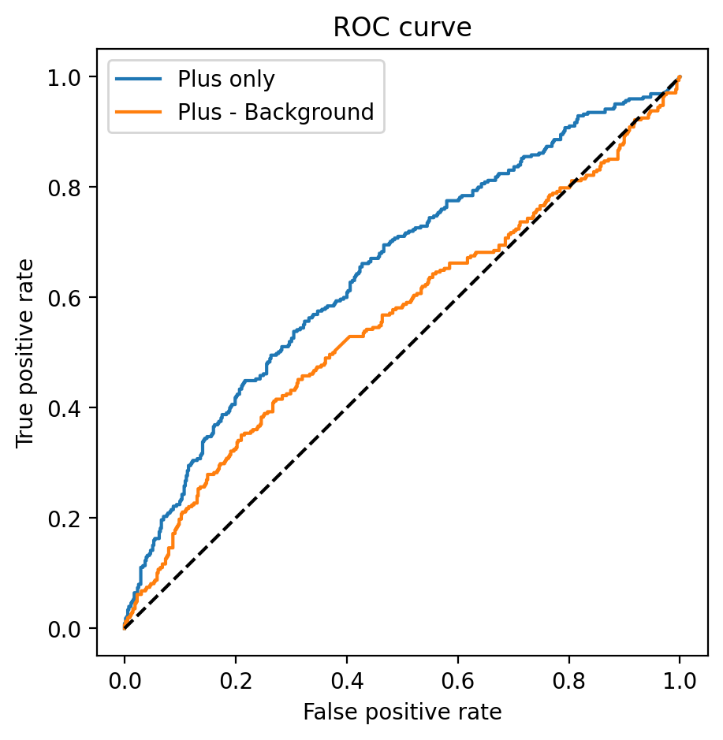


D. DMS/SEQ/*Arabidopsis*


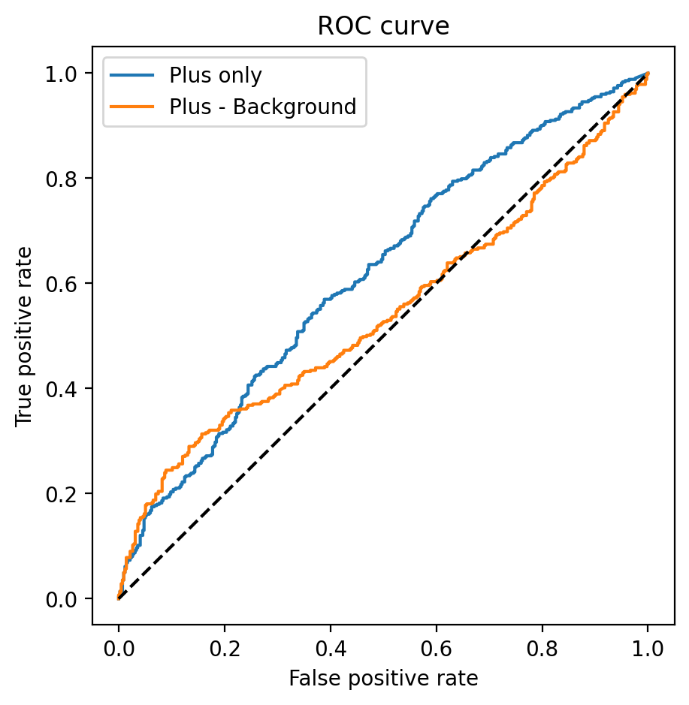


Figure S2 Comparison of ROC curves with and without background subtraction in chemical probing experiments

ROC curves were generated by mapping RNA chemical probing data to 18S/25S/28S rRNA. High reactivity was considered positive, and the dot-bracket data were considered true when the base was a dot. (**A**) Rice DMS reverse transcription stop profile. (**B**) Mouse SHAPE reverse transcription stop profile. (**C**) Yeast DMS reverse transcription stop profile. (**D**) *Arabidopsis* DMS reverse transcription stop profile. In each graph, the orange line represents the ROC curve using background data for reactivity calculation; the blue line represents the ROC curve without using background data for reactivity calculation. For the method of reactivity calculation, please refer to the Methods section.


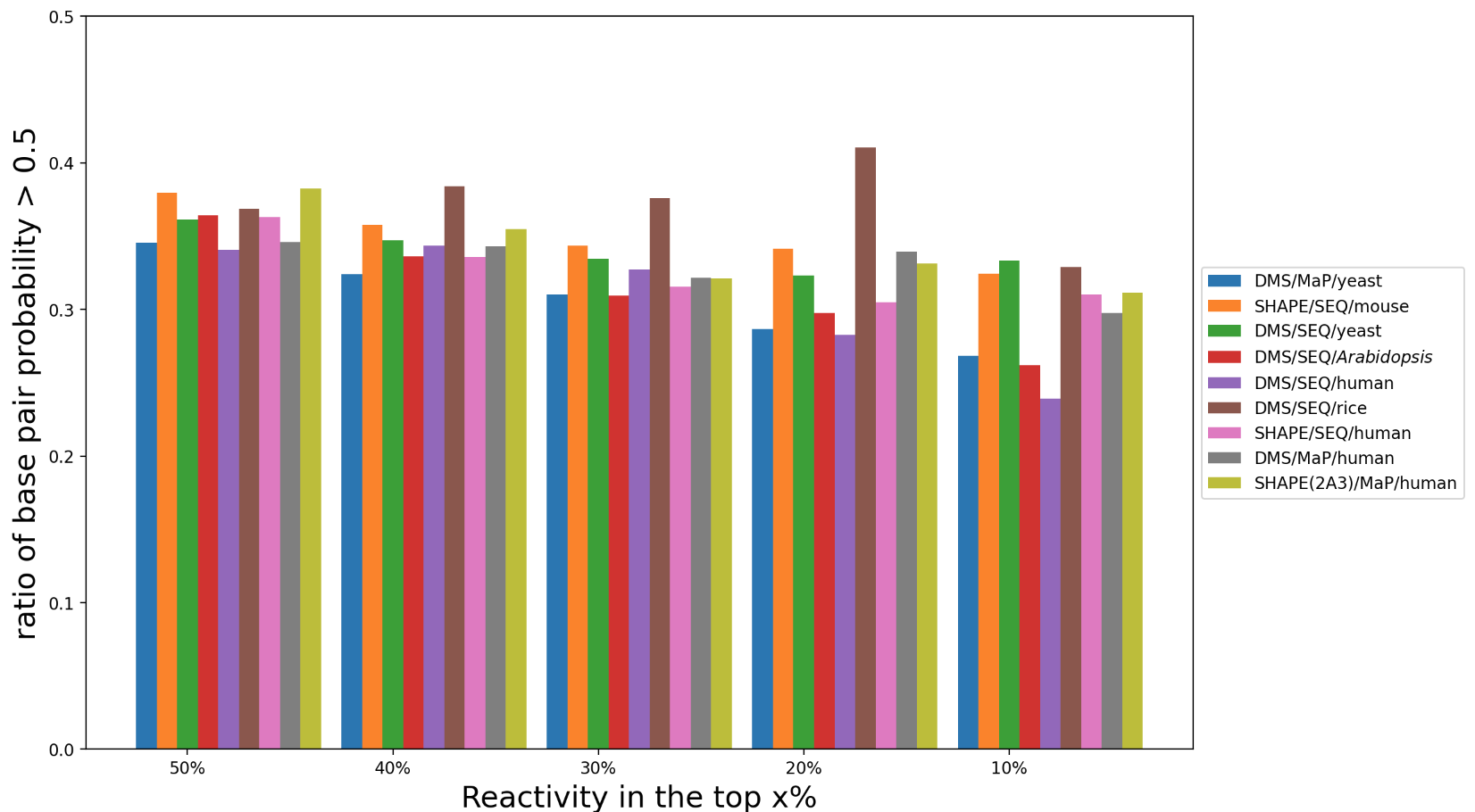


Figure S3. Base pair probabilities by reactivity range

The base pair probabilities of 18S rRNA were calculated for each reactivity range. Base pair probabilities were calculated using RNAfold. The horizontal axis shows the top x% of reactivity; the vertical axis shows the percentage of bases with the marginalized base pair probability of ≥0.5 in the top x% of reactivity.

Figure S4

A


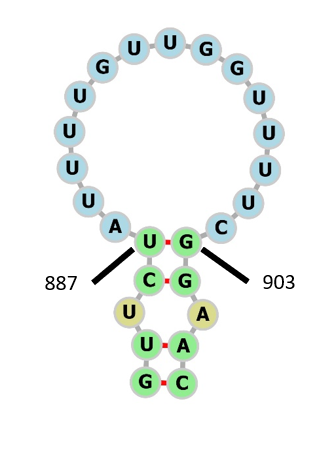


B


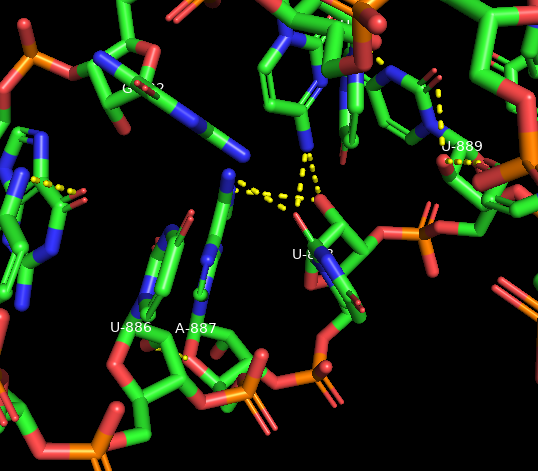


C


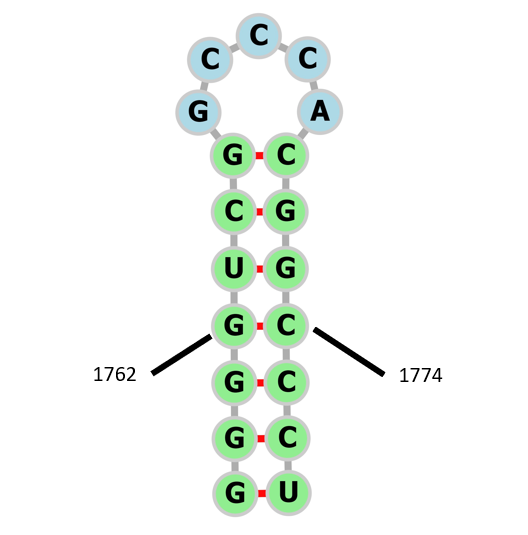


D


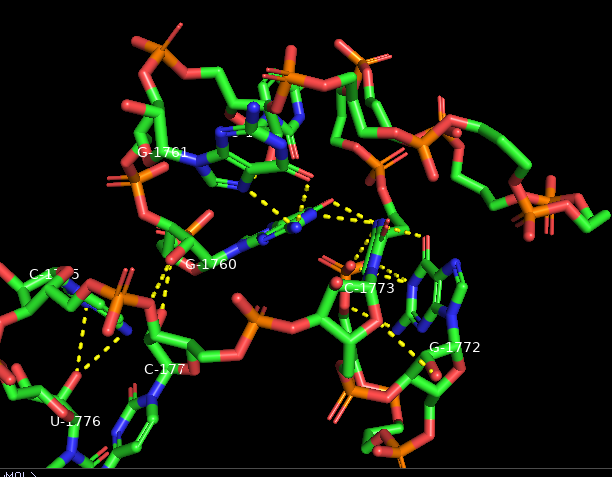


Figure S4. Secondary and tertiary structures of mouse 18S rRNA with high reactivity and base pairing predicted by RNAcentral and RNAfold

(**A**) Secondary structure around the 887U loop. 887U exhibits high reactivity (Table 4).

(**B**) Tertiary structure around the 887U loop. In the PDB ID: 7CPU sequence, 887U corresponds to 886U and is not involved in base pair formation.

(**C**) Secondary structure around the 1762G region. 1762G shows high reactivity (Table 4).

(**D**) Tertiary structure around the 1762G region. In the PDB ID: 7CPU sequence, 1762G corresponds to 1761G and is not involved in base pair formation.

Figure S5

A


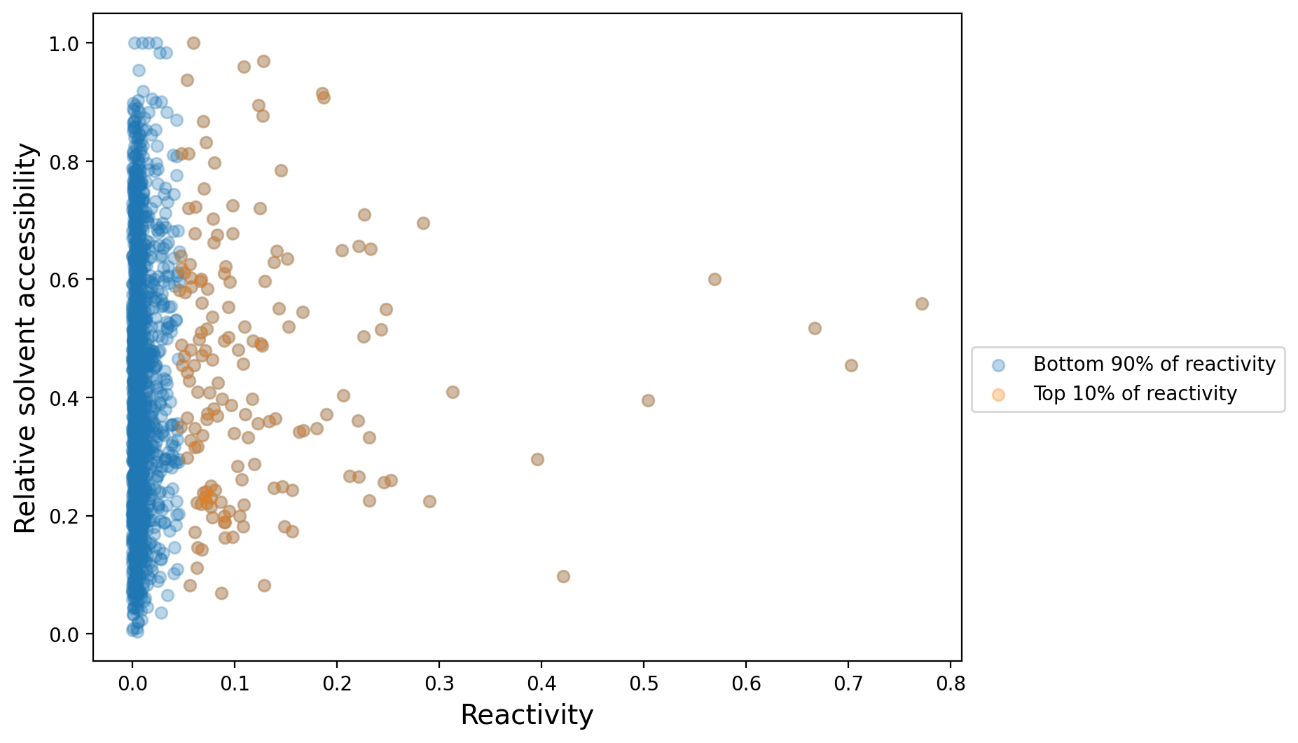


B


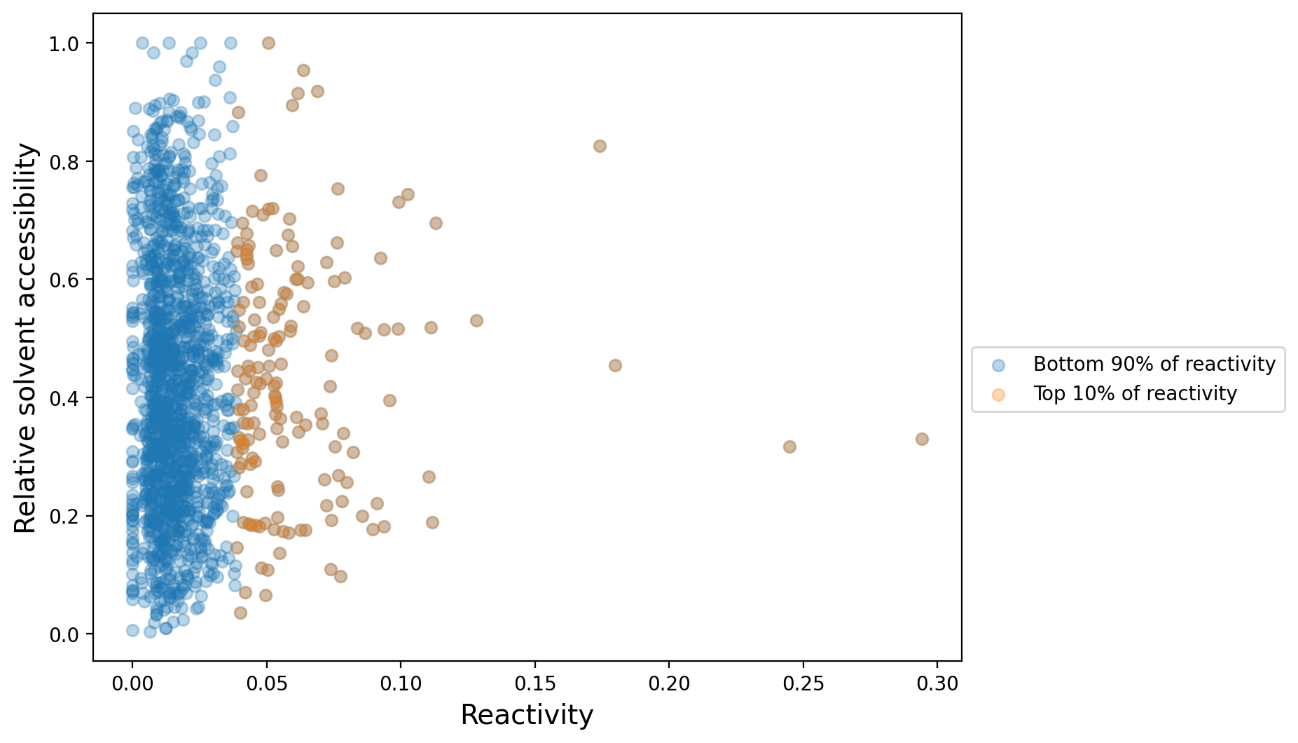


C


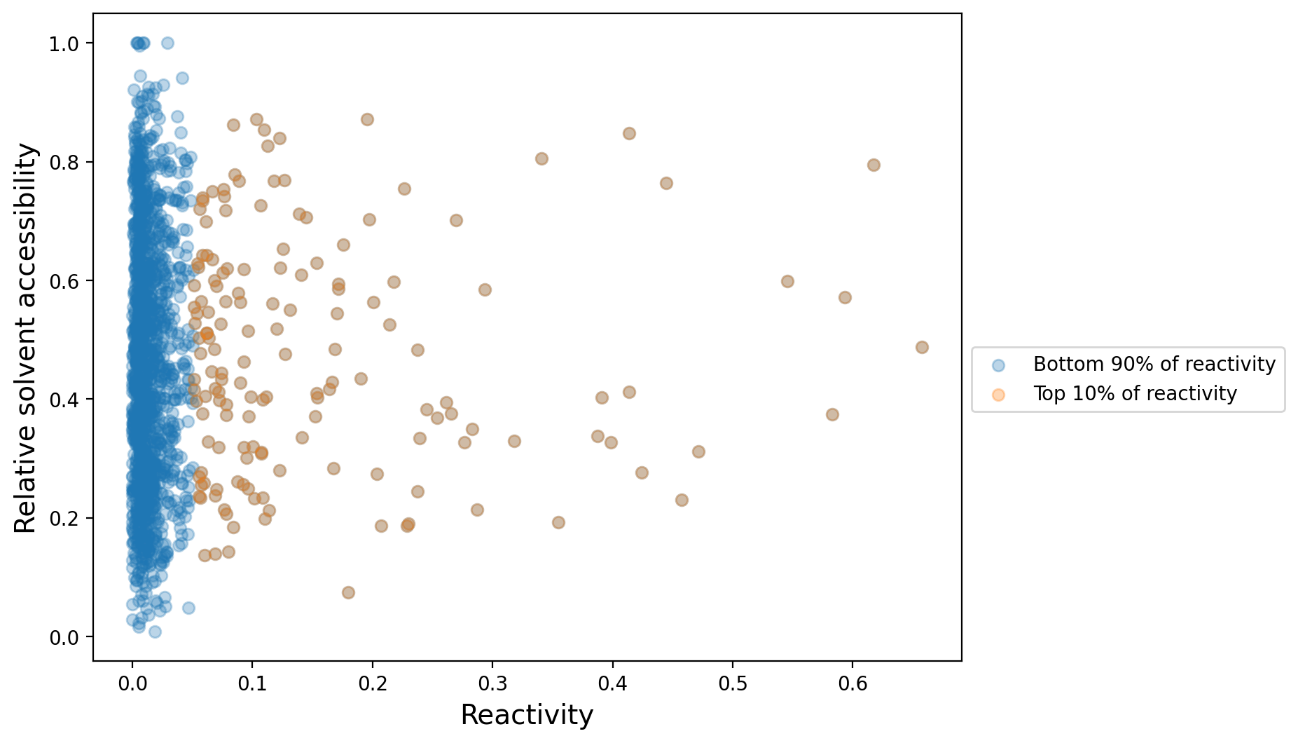


D


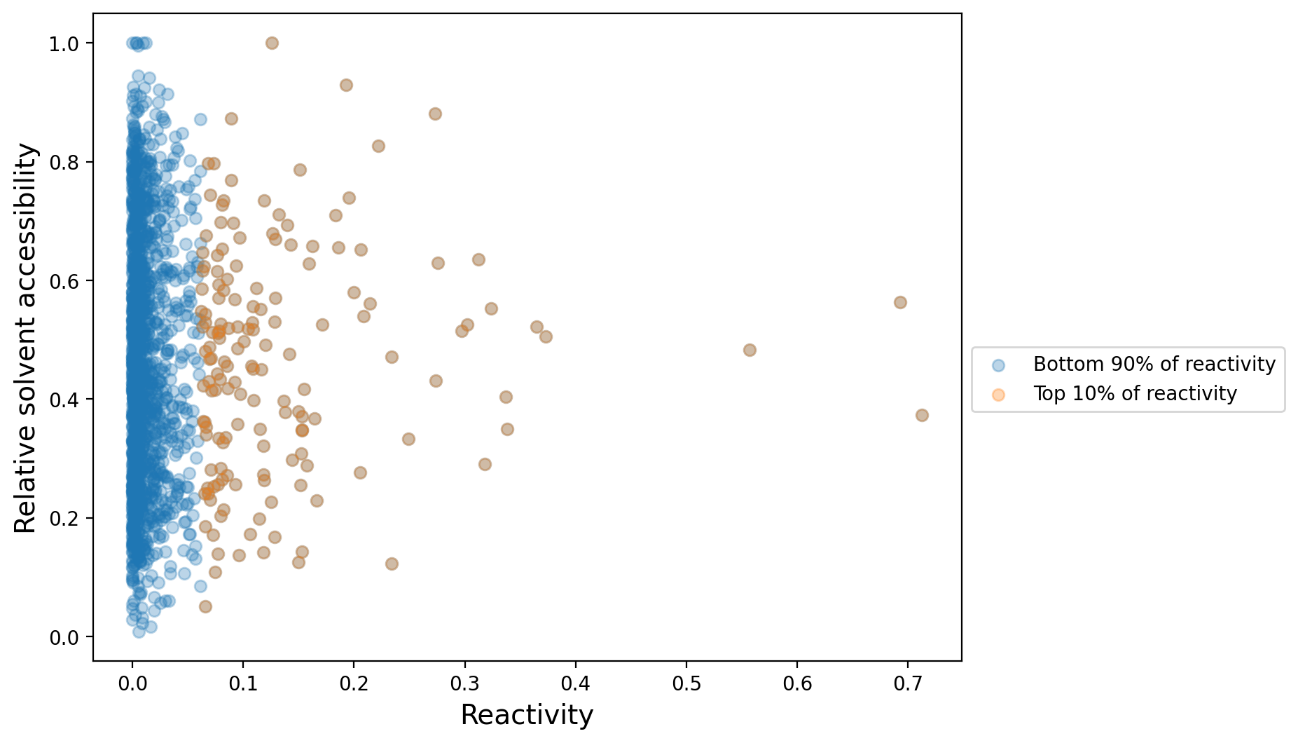


E


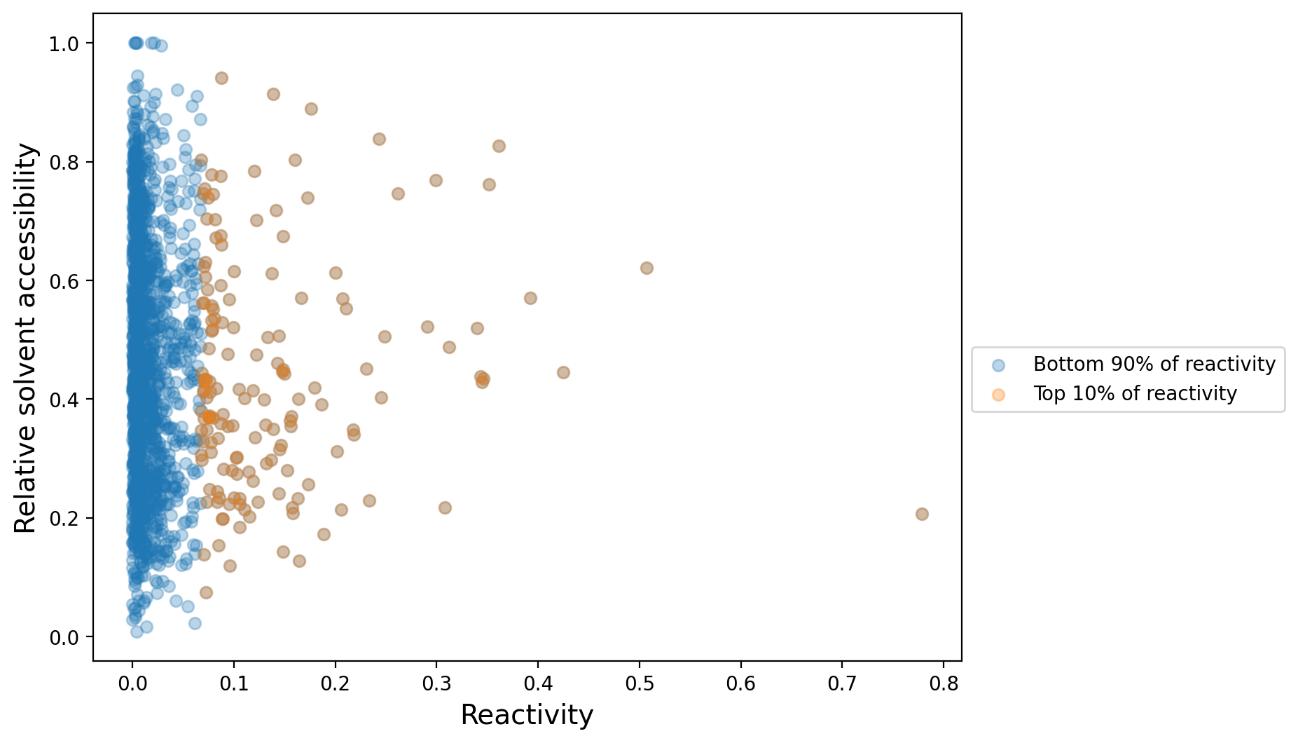


Figure S5. Relationship between solvent accessibility and reactivity

(A) The horizontal axis shows the reactivity of DMS/SEQ of yeast 18S rRNA, and the vertical axis shows the solvent accessibility of 18S rRNA (PDB ID: 8CCS).

(B) The horizontal axis shows the reactivity of DMS/MaP of yeast 18S rRNA, and the vertical axis shows the solvent accessibility of 18S rRNA (PDB ID: 8CCS).

(C) The horizontal axis shows the reactivity of DMS/SEQ of human 18S rRNA, and the vertical axis shows the solvent accessibility of 18S rRNA (PDB ID: 8JDK).

(D) The horizontal axis shows the reactivity of DMS/MaP of human 18S rRNA, and the vertical axis shows the solvent accessibility of 18S rRNA (PDB ID: 8JDK).

(E) The horizontal axis shows the reactivity of SHAPE/SEQ of human 18S rRNA, and the vertical axis shows the solvent accessibility of 18S rRNA (PDB ID: 8JDK).

The orange plot shows the bases with reactivity corresponding to the top 10%; the blue plot shows the bases with reactivity corresponding to the bottom 90%.


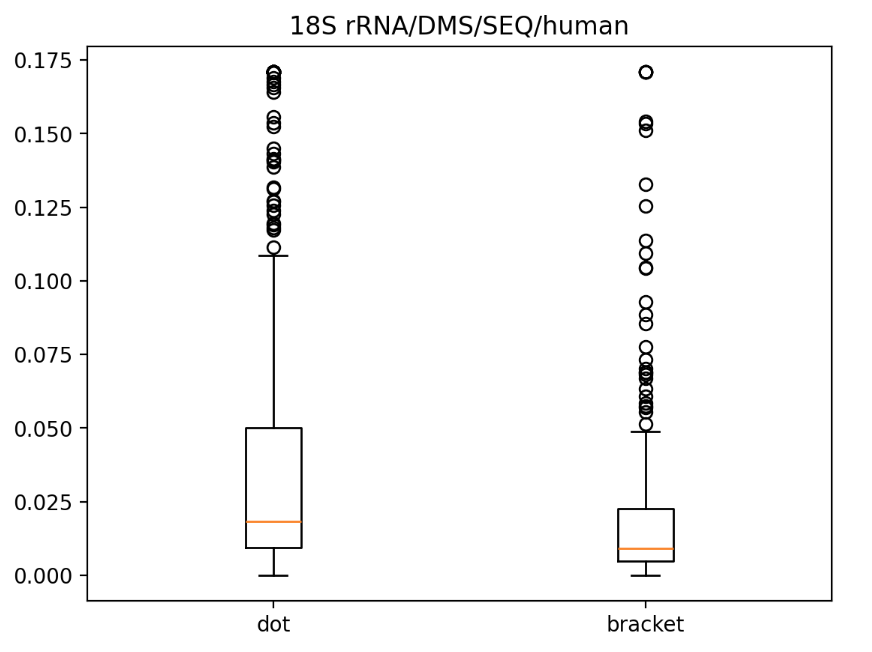


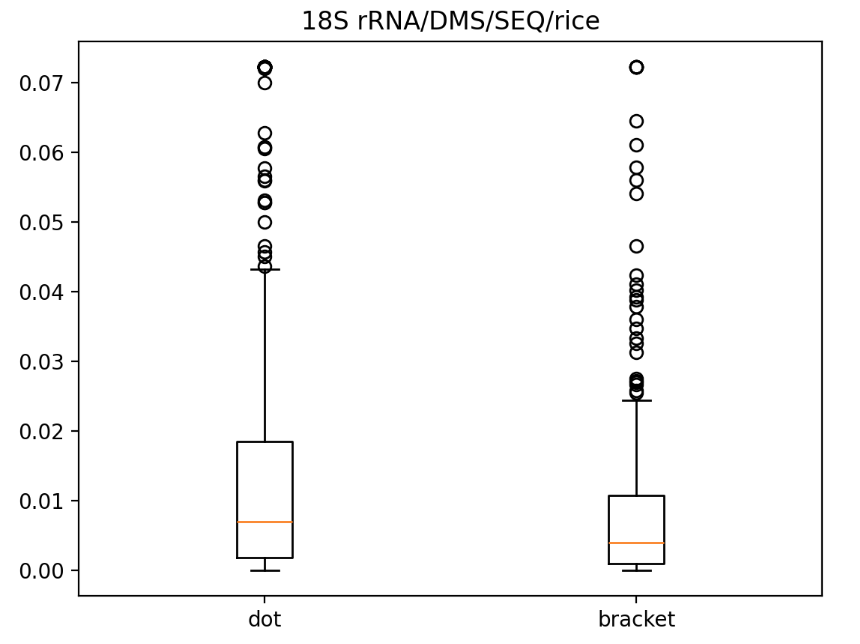


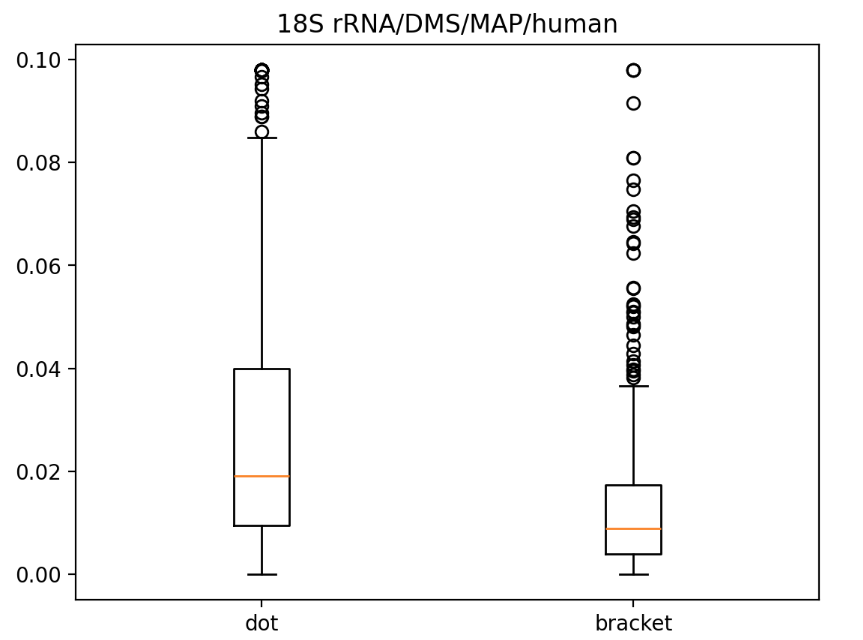


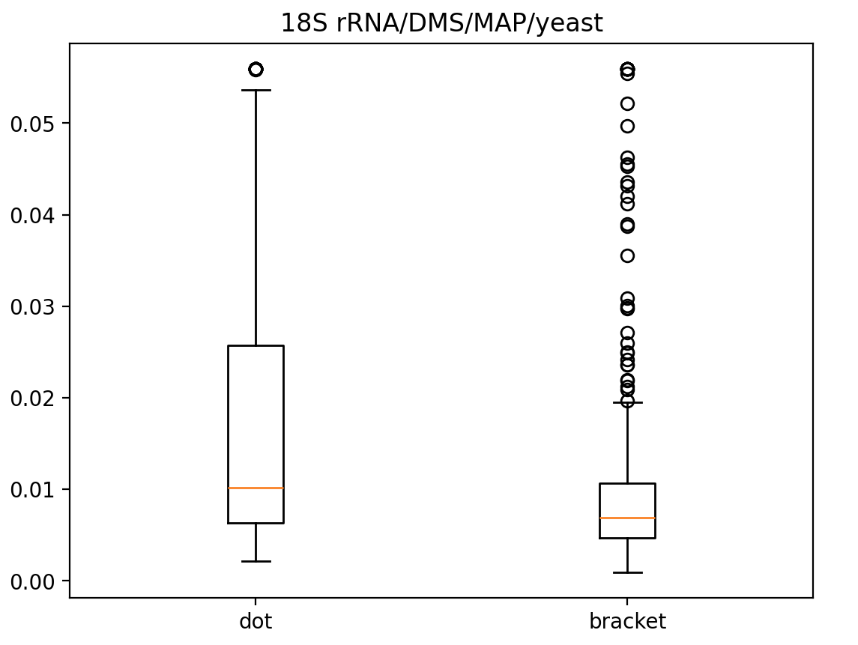


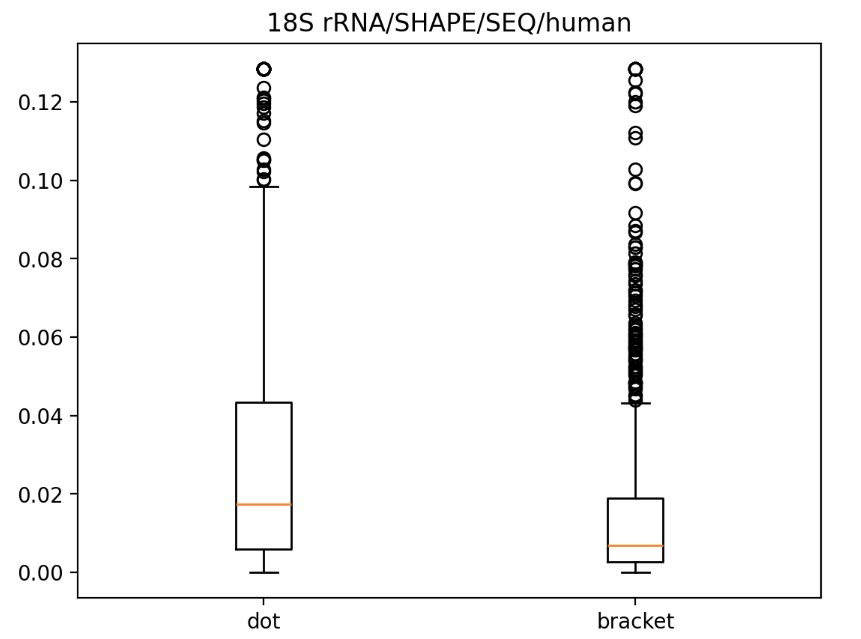


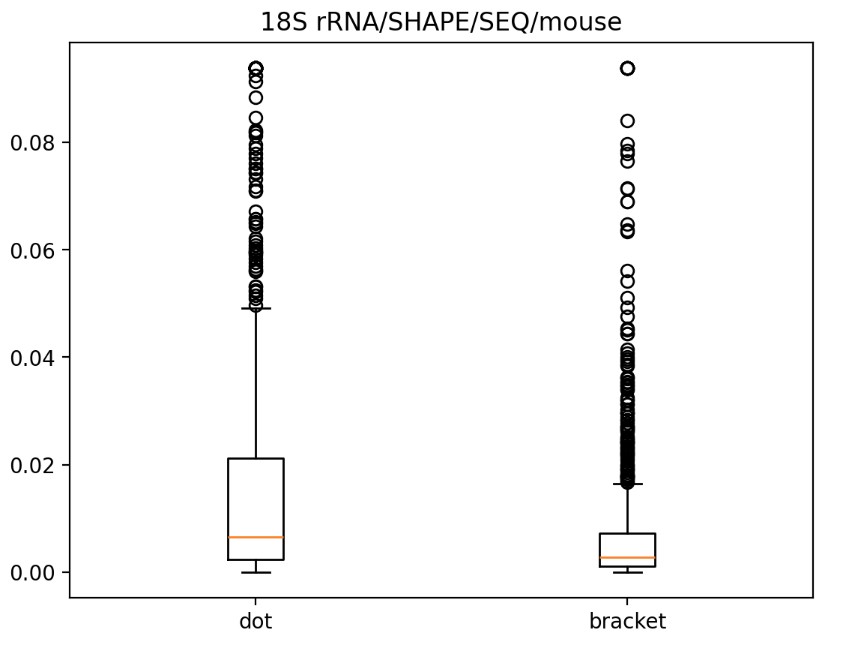


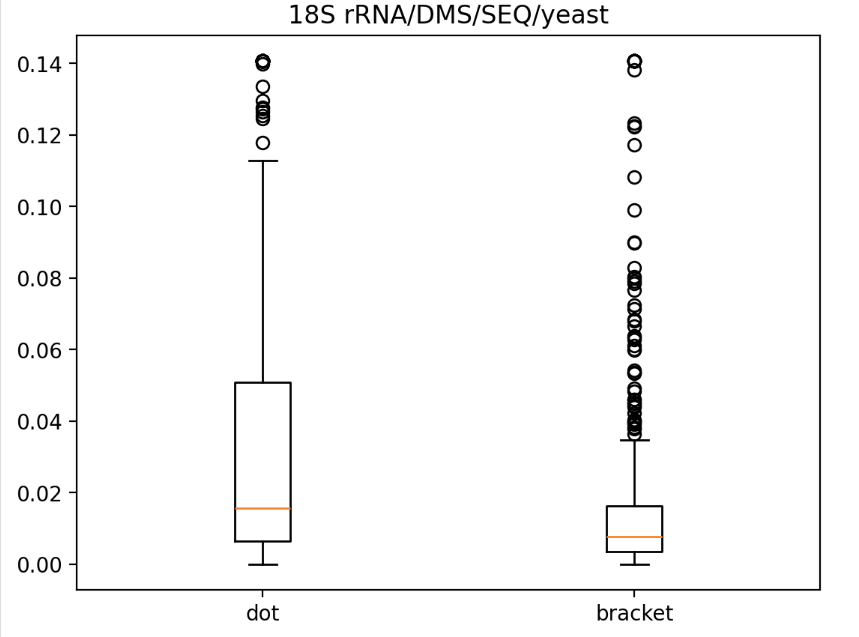


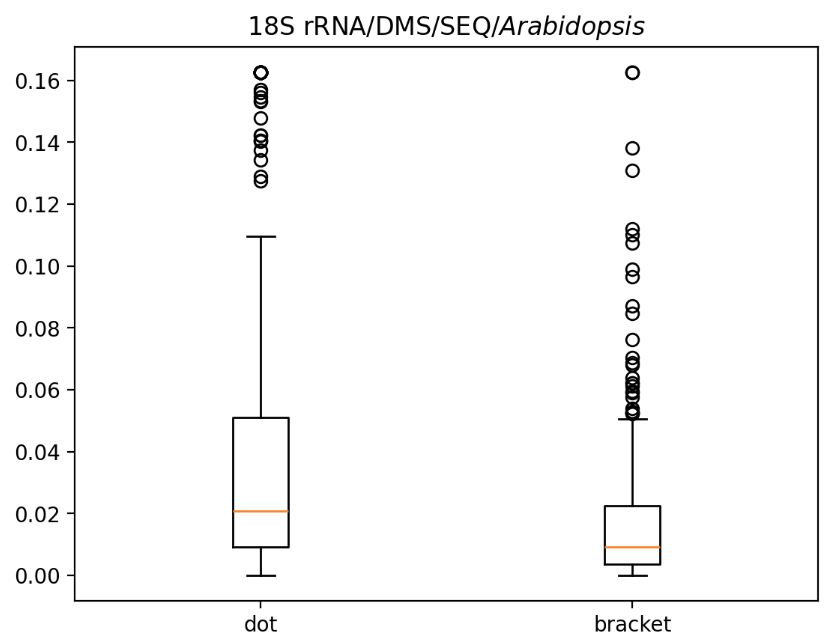


Figure S6

Box plots are presented to visualize the distribution of mutation/termination rates across paired and unpaired 18S rRNA structure. The vertical axis represents the mutation/termination rate, while the horizontal axis shows the dot-bracket notation derived from RNAcentral. Dots represent unpaired bases, and brackets represent paired bases. Due to the high peaks in mutation/termination rates, the top 5% of bases with the highest rates were rounded to the 95th percentile value. The orange line in the middle of the box plot represents the median, the bottom of the box represents the first quartile, the top of the box represents the third quartile, and the whiskers represent the most distant data points within 1.5 times the interquartile range of the box.
